# What is the impact of longer patient travel distances and times on perioperative outcomes following revision knee replacement: a retrospective observational study using data for England from Hospital Episode Statistics

**DOI:** 10.1136/bmjopen-2024-085201

**Published:** 2025-05-06

**Authors:** Alexander Handel Matthews, Jonathan Peter Evans, Jonathan Thomas Evans, Sarah Lamb, Andrew James Price, William Gray, Tim Briggs, Andrew D Toms

**Affiliations:** 1 Royal Devon and Exeter NHS Foundation Trust, Exeter, UK; 2 Getting It Right First Time Programme, NHS England, London, UK; 3 Department of Public Health and Sports Sciences, University of Exeter, Exeter, UK; 4 Nuffield Department of Orthopaedics, Rheumatology and Musculoskeletal Sciences, University of Oxford, Oxford, UK; 5 College of Medicine and Health, University of Exeter, Exeter, UK; 6 Nuffield Orthopaedic Centre, Oxford, UK; 7 Royal National Orthopaedic Hospital, London, UK

**Keywords:** Knee, PUBLIC HEALTH, Health Services

## Abstract

**Abstract:**

**Objectives:**

Patients undergoing revision total knee replacement (RevKR) surgery often have difficulties mobilising and increasingly rely on family support. Evolving practice in England aims to manage these patients in specialised centres with the intention of improving outcomes. This practice will result in longer travel distances and times in this frailer group of patients. We want to examine the types of distances and travel times patients can be expected to travel for this complex orthopaedic surgery and to explore concerns of how these impact patient outcomes.

**Design:**

Retrospective observational study from the Hospital Episode Statistics. Multivariable adjusted logistic regression models were used to investigate the relationship between patient travel distances and times with perioperative outcomes.

**Setting:**

Patients presenting to tertiary referral centres between 1 January 2016 and 31 December 2019. A tertiary referral centre was defined as a trust performing >49 revisions in the year prior.

**Participants:**

Adult patients undergoing RevKR procedures for any reason between 1 January 2016 ando 31 December 2019.

**Exposure:**

The shortest patient level travel distance and time was calculated using the Department of Health Journey Time Statistics using Transport Accessibility and Connectivity Calculator software and Dijkstra’s algorithm.

**Main outcome measures:**

The primary outcome is emergency readmission within 30 days. Secondary outcomes are mortality within 90 days and length of inpatient stay.

**Results:**

6880 patients underwent RevKR at 36 tertiary referral centres. There was a weak correlation between social deprivation and travel distance, with patients from the most deprived areas travelling longer distances. Overall, 30-day readmission was not statistically associated with longer driving distance (OR 1.00 95% CI 0.99 to 1.02) or peak driving times (OR 1.00 95% CI 0.99 to 1.01).

**Conclusions:**

There was no association between increasing travel distance and time on perioperative outcomes for RevKR patients.

**STRENGTHS AND LIMITATIONS OF THIS STUDY**

- This study is one of the largest studies in the literature investigating outcomes following revision knee replacement.
- This data reflect revision knee replacement procedures undertaken across different geographical areas of England.
- Owing to differences in the coverage of Hospital Episode Statistics, procedures in hospitals outside of England were not included in this analysis.
- Clinical coding practice is known to vary across trusts, with some trusts more consistent in coding than others, which may have created some bias in the model estimates.
- This analysis only reports travel times for patients with access to their own transport and does not consider times for those patients using public transport.

## Introduction

 Primary knee replacement is a successful procedure that improves quality of life for the majority of patients.^1^ However, at 10 years following a primary knee replacement, about 3.5% of patients will have undergone a revision surgery.^2^ The majority of these procedures are carried out due to infection or polyethylene wear of the implant.^3^ A failed primary knee replacement represents a life-changing transition point where individuals are likely to suffer from pain, reduced mobility as well as dependency on family members.^4^ Patients often face multistep surgery with longer hospital length of stays and higher complication rates.^5 6^

The Getting It Right First Time programme orthopaedic National Report was published in 2015.^7^ A key recommendation was the centralisation of complex orthopaedic surgery, including revision knee surgery, to specialist centres with the aim of improved patient outcomes. Consequently, revision total knee replacement (RevKR) surgery in England has evolved into a regional network service model.^8^ All hospitals performing RevKR form a network in the respective regions. Less specialist hospitals, defined by lower annual case volume thresholds, are encouraged to discuss and sometimes refer their caseload to more specialist centres. Several studies based on large revision hip and knee registries have suggested this model carries a lower failure rate defined by the need for further revision surgery.^9^,^11^ Early evidence has suggested reduced early failure rates through the adoption of revision knee networks.^12^

However, for some patients, this approach to managing patients is inevitably associated with increasing travel distances between patients’ homes and their treating hospital. Travel distance has been shown to be an important factor in patient choice when selecting a surgeon for joint replacement surgery. It may be even more important for those awaiting revision joint replacement surgery as these patients struggle with mobility, may be unable to drive and may be more reliant on family members.^4^ Evidence suggests that patients considering joint replacement are prepared to travel longer distances to obtain the best possible outcomes. A requisite in making such a decision requires data on outcomes of patients travelling greater distances. Patients travel longer distances have been found to have higher readmission rates and higher mortality rates when undergoing other types of specialised surgery.^13^ The pick-up rate of early complications, avoiding the need for readmission, may be less in areas further away from the main treatment centre. There is also concern that patients required to travel greater distances are more likely to be readmitted to a different hospital than that where surgery was undertaken, resulting in clinical decisions that do not incorporate the primary surgeon and so potentially leading to poorer outcomes.^14^ There is an absence of evidence in the literature to support or refute this argument in the context of patients undergoing RevKR. Therefore, the aim of this paper is to investigate the relationship between longer patient travel distances and perioperative outcomes following RevKR performed in high-volume tertiary referral centres.

## Methods

### Design

This study is a retrospective data analysis of observational data from the Hospital Episode Statistics (HES) and Office for National Statistics (ONS) databases. HES data are collected by NHS England for all patients treated at NHS hospitals in England and those treated at private hospitals where treatment was funded by the NHS. This study complies with the recommended reporting guidelines when using HES data^15^ and the Strengthening of Reporting of Observational studies in Epidemiology guidelines.^16^

The analysis and presentation of data follows current NHS England guidance for the use of HES data for research purposes^17^ and is anonymised to the level required by ISB1523 Anonymisation Standard for Publishing Health and Social Care Data.^18^ The HES data were linked at a patient level to data from the ONS on deaths and date of death, which allowed the identification of patients who had died after their surgery. Linkage was achieved using a unique pseudonymised patient identifier using a previously validated methodology.^19^

Patient travel distances were calculated using the Journey Time Statistics reference document produced by the UK Department of Transport which modelled theoretical journey times between known centroids of lower layer super output areas (LSOAs) of residence and NHS hospital sites.^20^ Please refer to online supplemental material S1 for Journey Times Statistics reference document.

### Population

An RevKR procedure was defined as a permanent removal or exchange of knee arthroplasty components. This includes a revision of a total knee replacement and a conversion of a unicondylar knee replacement to a total knee replacement. Secondary patellar resurfacing was not included as this represents a simple revision procedure, one that can be carried out in most nonspecialised hospitals. All patients aged ≥18 years who underwent a RevKR in a high-volume trust between 1 January 2016 and 31 December 2019 were included in the study population. A high-volume trust was classified as a centre performing >49 revisions per year. This revision volume threshold for classification represents that proposed by the British Association for Surgeons of the Knee Revision Knee Working Group and is a mandatory requirement for all highly specialist centres co-ordinating regional networks.^21^ As such, centres attaining this threshold are more likely to represent tertiary referral centres where the stratification of more complex work will take place. Annual case volume at each trust was defined as the number of revision cases conducted in the year prior to the index procedure. This measure was preferred over a simple calculation of average annual volume as it accounts for recent experience at the point of surgery. The Office for Population Censuses and Surveys’ Classification of Interventions and Procedures version 4 codes used to identify RevKR procedures are detailed in online supplemental material S2. Since laterality was needed to identify re-revisions, patients were excluded where the procedure laterality was not specified. The flow of patients, with numbers excluded at each point, is summarised in online supplemental material S3. To manage population heterogeneity, data were extracted for the period from 1 April 2011 to 31 December 2019, and only the first revision for a specific side of the body record in this time period was included.^22^ Thus, any early revisions on the same side of the body in the 4 years and 9 months preceding the start of the study period were identified and these patients excluded from the study. This aims to exclude the early revision knee replacement failures which have been shown to represent catastrophic failures potentially skewing our results.^22^ We included revisions for infection as, despite these representing a more variable patient group, the presence of infection was thought to be unrelated to how far a patient lives from a specialised referral centre.

### Exposure variable

Travel distances and times were calculated between a patient’s LSOA and the postal codes for the treating hospitals. LSOAs are determined by the ONS and are designed for the reporting of small area statistics. Public transport and highways data for England were used to create theoretical journey distances and times from origins to destinations. A network of journey distances and times from origins to destinations was produced using a software package called Transport Accessibility and Connectivity Calculator. The Dijkstra’s algorithm calculated the shortest route between these points. Data linkage between the HES/ONS dataset and the travel times dataset was achieved using two shared data fields: LSOA and hospital site. The resulting travel distances and/or times for each patient were analysed as continuous variables. Three exposure variables were used. Straight line travel distance represented the distance ‘as the crow flies’ between a patient’s LSOA and treating hospital. Off-peak driving distance represented the shortest driving distance between a patient’s LSOA and treating hospital. Finally, peak driving times were calculated using average traffic speeds between 7:00 and 10:00 hours for the shortest possible road route between a patient’s LSOA and treating hospital. These three variables were used to account for variation in travel infrastructure between rural and urban areas and to attribute more meaningful results for patients.

### Covariates and cluster variable

The following groups of known or potential confounding variables were chosen a priori for inclusion in our multivariable logistic regression modelling:

#### Patient factors

Patient factors included age in years (continuous), sex (male/female). Health comorbidity was quantified using the Hospital Frailty Risk Score (HFRS). HFRS identifies frailty based on the occurrence of any of 109 International Statistical Classification of Diseases and Related Health Problems, 10th revision (ICD-10) codes used during any hospital admissions in the 2 years prior to, and for, the index admission. Deprivation was measured using the Index of Multiple Deprivation (IMD).^23^ The IMD gives the LSOA where the patient lives a score based on a range of measures of deprivation. IMD was analysed as a continuous variable.

#### Clinical factors

Clinical factors is defined by the presence or absence of infection as the primary indication for RevKR. This was identified from the ICD-10 and Related Health Problems codes used during the admission.

#### Surgical factors

Surgeon and hospital volume (both continuous) were defined as the number of RevKRs performed by a consultant or hospital in the 365 days prior to each index procedure across the entire cohort. This was calculated before any exclusion criteria were applied.

#### Temporal factors

Financial year of procedure (2015/2016, 2016/2017, 2017/2018, 2018/2019, 2019/2020).

#### Hospital provider

Clustering of patients by hospital provider was initially modelled using random effects. However, despite variability between hospital providers with primary and secondary outcomes, instability in the model estimates was observed. To address the possibility of clustering at this level, a fixed effects model was adopted with hospital provider as a covariate.

### Outcomes

The primary outcome was emergency readmission within 30 days of discharge from the index surgical hospital. Readmission in this early period is very likely related to a complication of the surgical procedure. It has been used as a marker of perioperative outcomes in similar studies investigating the relationship between patient travel distance and outcomes following surgery.^13^

Secondary outcomes were 90-day all-cause mortality, identified using linked data from Civil Registrations (Mortality) dataset.

Inpatient length of hospital stay was attributed from continuous inpatient spells, which is the preferred estimate of length of stay. This refers to the length of the first stay after the operation regardless of any transfers across providers. The median length of stay was calculated after visually inspecting the distribution, and this was dichotomised into prolonged length of stay if longer than the median stay.

### Statistical analyses

Data were extracted from a secure, encrypted server controlled by NHS England. Data were analysed within a secure, encrypted environment using standard statistical software: R Studio V.2023.09.1+494 (Boston, Massachusetts, USA). The R code and packages used are included in online supplemental material S4.

Missing data were managed according to their extent and relevance to the aims of this study. Age and IMD score were imputed for the small number of missing cases using the mean of the entire study cohort. Given the central role of LSOA in estimating travel distances and times and fewer than 5% of cases with missing data, these cases were excluded to avoid the introduction of bias. Following data linkage between the HES/ONS dataset and the travel times dataset, approximately 36% (n=5838) of cases did not match. Multiple imputation was performed using predictive mean matching based on the entire cohort of patients with the following predictors: age, sex, HFRS score, IMD score, hospital provider code, hospital volume and surgeon volume. Dependent variables including readmission at 30 days, mortality at 90 days and length of stay were also used in the imputation following a recommended approach using predictive mean matching.^24^ A total of five imputations were randomly chosen and subsequent regression analyses were performed.^25^ Imputed data are shown in online supplemental material S5.

Patient travel distances were categorised into quintiles for interpretation of baseline demographics and clinical characteristics. Subsequent analysis of travel distances and times was performed as continuous variables. Spearman’s rank correlation was performed to investigate the relationship between IMD score and patient age with travel distances.

Straight line travel distance was modelled with restricted cubic splines to allow for the non-linear effects when testing the association with the primary outcome. All exposures were modelled with restricted cubic splines to allow for the non-linear effects when testing the association with prolonged length of stay. The Akaike information criterion was used to select the most parsimonious specification of restricted cubic splines using the final adjusted model. Fixed effects logistic regression models were used for the outcomes of readmission at 30 days, mortality at 90 days and prolonged length of stay. Where implemented, the use of splines was used to create figures depicting the association between travel distance or times and probability of the outcomes. Only adjusted spline models were used to depict these associations. All covariates were included in the adjusted models. Multicollinearity was assessed using eigenvalues, variance inflation factors and by examination of model parameter estimates with the unadjusted model. ORs with 95% CIs and associated p values were reported. A p<0.05 was taken to indicate statistical significance.

### Public and patient involvement

The study’s chief investigator (ADT) led the James Lind Alliance ‘Revision Knee Replacement’ priority setting partnership. This group of patients, carers and healthcare professionals identified the need to investigate the best way of organising revision knee replacement surgery to improve patient outcomes as 1 of their top 10 research questions. Patients were, therefore, directly involved in the development of the study’s aims and objectives. The results of the study will be disseminated to the members of this group prior to publication.

## Results

### Overview of results

**Figure 1:**
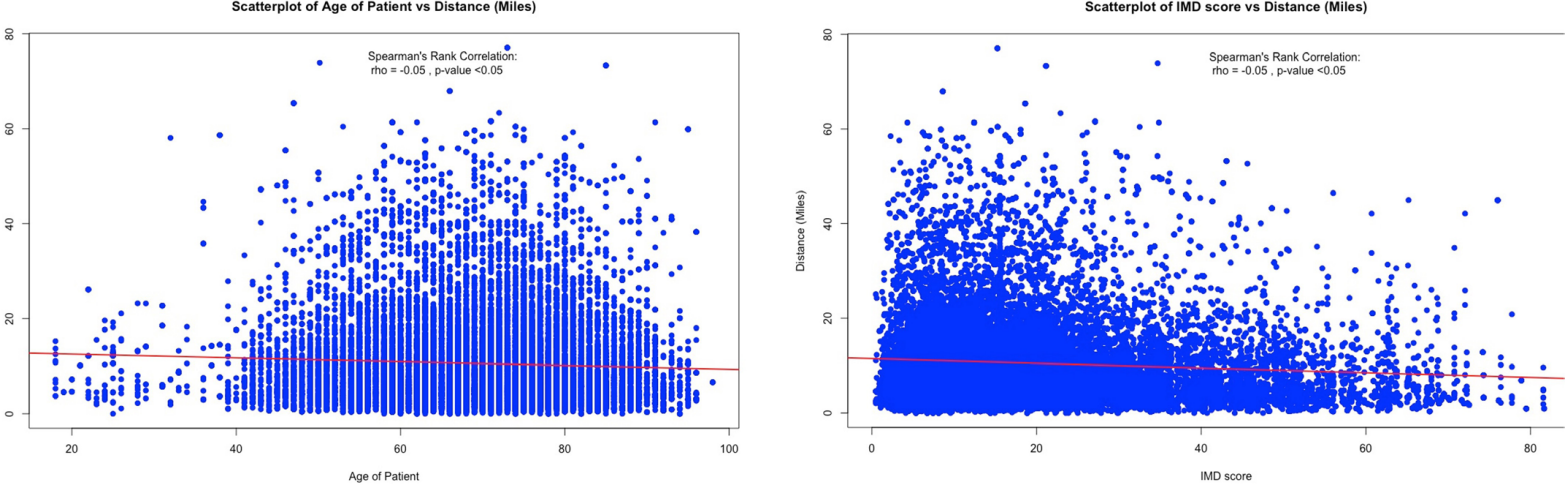
(Left) Scatterplot showing correlation between patient age and travel distance. Red line represents linear regression trend. Spearman’s rank correlation is presented in the chart. (Right) Scatterplot showing correlation between social deprivation and patient travel distance. Red line represents linear regression trend. Spearman’s rank correlation is presented in the chart. IMD, Index of Multiple Deprivation.

A total of 16 736 patients met the inclusion criteria. Excluding missing LSOA data (n=171), 16 565 patients were included in the analysis. Following data linkage with the Department of Transport journey times statistics, 10 727 patients had complete data linkage and data were imputed for the remaining 5838 (35.2%). Of the 16 565 patients, 41.5% (n=6880) presented to a tertiary referral centre and these data formed our analysis cohort. Patients were operated on across 181 hospital sites and 38 hospital trust providers. The baseline demographic and clinical characteristics of the patients were broadly similar between quintiles of straight-line travel distance (table 1). Higher hospital volumes were seen in patients travelling longer distances. Figure 1 shows that straight line travel distance was weakly correlated with age (r=−0.05, p<0.05) and social deprivation (r=−0.05, p<0.05). Older patients were less likely to travel farther distances. Patients from the least deprived areas travelled shorter distances.

**Table 1:** Baseline patient demographics and clinical characteristics stratified by travel distance quintiles from first imputed dataset

	Travel distance quintile				
	1	2	3	4	5
Distance (miles)	2.09 (1.35 to 2.75)	4.42 (3.91 to 5.00)	7.08 (6.34 to 7.99)	11.39 (10.11 to 12.74)	22.42 (18.09 to 32.19)
Driving time (minutes)	13 (9.3 to 17)	20.45 (17 to 25)	26.30 (21.98 to 31.13)	34.10 (29.68 to 40.20)	52.05 (42.68 to 66.83)
Number of patients	1376	1376	1376	1376	1376
Tertiary providers	37 (97.37%)	38 (100%)	36 (94.74%)	35 (92.11%)	37 (97.37%)
Age mean (SD)	69.71 (10.81)	69.96 (10.71)	69.66 (10.92)	68.84 (11.01)	68.58 (10.75)
Female sex	762 (55.38%)	768 (55.81%)	729 (52.98%)	722 (52.47%)	734 (53.34%)
HFRS none	647 (47.02%)	620 (45.06%)	614 (44.62%)	666 (48.40%)	676 (49.13%)
HFRS mild	438 (31.83%)	474 (34.45%)	485 (35.25%)	465 (33.79%)	433 (31.47%)
HFRS moderate	241 (17.51%)	236 (17.15%)	243 (17.66%)	198 (14.39%)	230 (16.72%)
HFRS severe	50 (3.63%)	46 (3.34%)	34 (2.47%)	47 (3.42%)	37 (2.69%)
Infection present	314 (22.82%)	331 (24.06%)	310 (22.53%)	334 (24.27%)	355 (25.80%)
Surgeon volume	7 (3 to 13)	7 (3 to 13)	8 (3 to 15)	8 (3 to 16)	9 (4 to 17)
Hospital volume	73 (60 to 87)	74 (60 to 89)	79 (63 to 97)	79 (63 to 99)	85 (68.75 to 112)
IMD score	16.44 (8.73 to 28.67)	14.30 (7.96 to 24.57)	14.50 (8.47 to 21.36)	14.83 (9.23 to 21.74)	14.752 (8.78 to 21.45)
Year 2015/2016	104 (7.56%)	94 (6.83%)	94 (6.83%)	89 (6.47%)	92 (6.69%)
Year 2016/2017	383 (27.83%)	354 (25.73%)	348 (25.29%)	338 (24.56%)	353 (25.65%)
Year 2017/2018	384 (27.91%)	365 (26.53%)	339 (24.64%)	360 (26.16%)	336 (24.42%)
Year 2018/2019	269 (19.55%)	325 (23.62%)	347 (25.22%)	354 (25.73%)	339 (24.64%)
Year 2019/2020	236 (17.15%)	238 (17.30%)	248 (18.02%)	235 (17.08%)	256 (18.60%)

HFRS, Hospital Frailty Risk Score; IMD, Index of Multiple Deprivation.

### Outcomes

The primary and secondary outcomes are summarised in table 2.

**Table 2:** Adjusted pooled multivariable logistic regression showing ORs for primary and secondary outcomes by exposure variables

	Straight line travel distance (OR, 95% CI)	Travel distance by shortest road route (OR, 95% CI)	Peak travel times by shortest road route (OR, 95% CI)
Readmission with 30 days	Figure 2	1.00* (0.99 to 1.02), p=0.81	1.00* (0.99 to 1.01), p=0.69
90-day mortality	1.00* (0.98 to 1.02), p=0.87	1.00* (0.99 to 1.01), p=0.86	1.00* (0.99 to 1.01), p=0.89
Prolonged length of stay	Figure 3	Figure 4	Figure 5

* ORs have been adjusted for patient age, sex, HFRS score,IMD, annual surgical volume, hospital provider

CI, Confidence Interval; HFRS, Hospital Frailty Risk Score; IMD, Index of Multiple Deprivation; OR, Odds Ratio.

**Figure 2:**
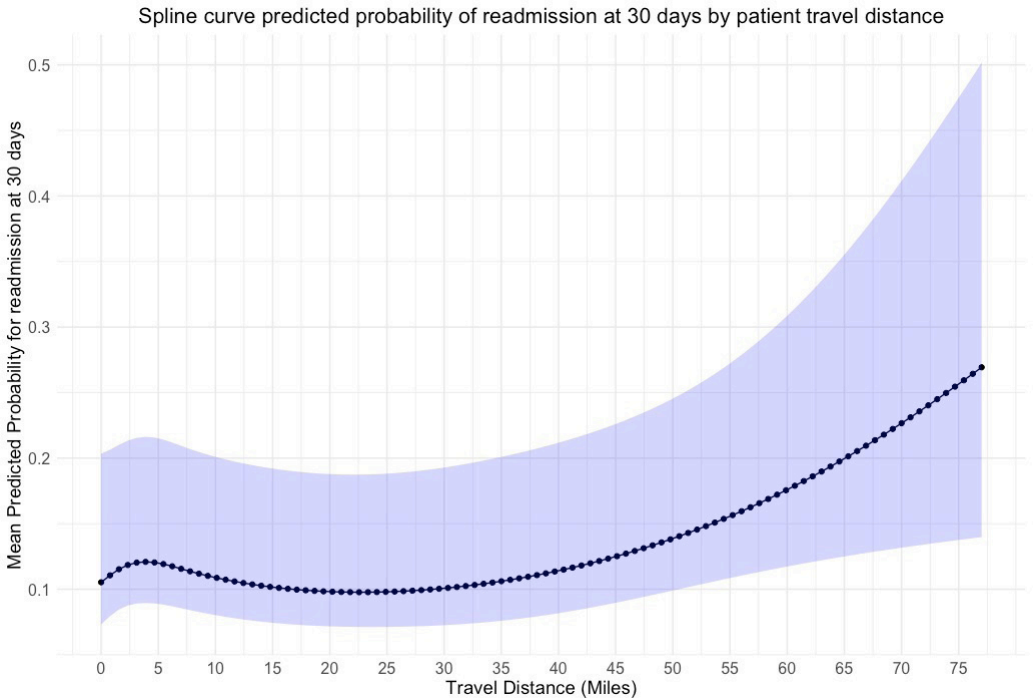
Predicted probability of emergency readmission at 30 days by straight line patient travel distance from hospital after RevKR. A fixed effects multivariable logistic regression model using 3 knots at 5%, 50% and 95% centiles of mean unit volume. 95% CIs represented by blue shaded line. RevKR, revision total knee replacement.

**Figure 3:**
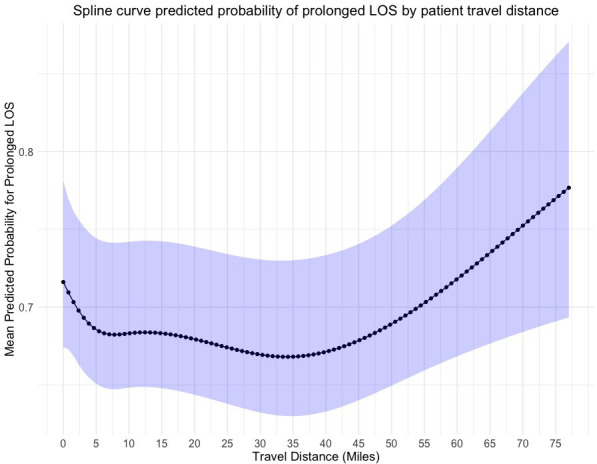
Predicted probability of prolonged length of inpatient stay by patient straight line travel distance from hospital after RevKR. A fixed effects multivariable logistic regression model using 4 knots at 5%, 35%, 65% and 95% centiles of mean unit volume. 95% CIs represented by blue shaded line. LOS, length of stay; RevKR, revision total knee replacement.

The observed rate of readmission at 30 days was 8.3% (568/6880). There was a negative association between higher straight line travel distances and emergency readmission at 30 days (figure 2). However, wide CIs precluded statistical inferences. In addition, higher travel distance by road and longer drive times were not associated with statistically worse readmission rates at 30 days. The rate of mortality at 90 days was only 3.2% (217/6880). No statistically significant relationship was observed between the distance a patient travels by road or the time a patient spends travelling at peak driving times with rates of mortality at 90 days. 49.7% (3421/6880) of patients reported hospital stays of more than 5 days. Following adjustment of confounding factors, we observed no associations between prolonged length of stay and patient travel distance (figures3^5^).

**Figure 4:**
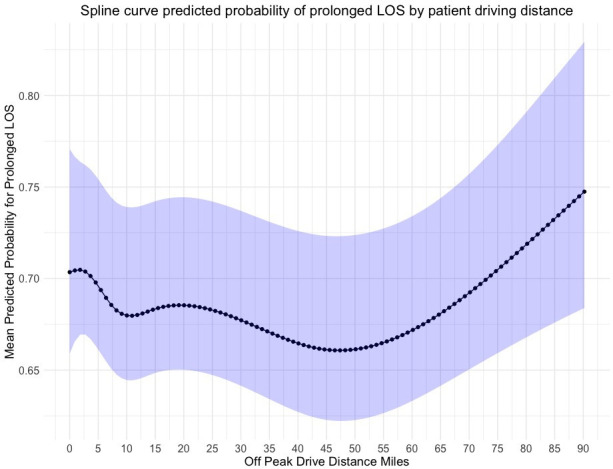
Predicted probability of prolonged length of inpatient stay by patient driving distance from hospital after RevKR. A fixed effects multivariable logistic regression model using 4 knots at 5%, 35%, 65% and 95% centiles of mean unit volume. 95% CIs represented by blue shaded line. LOS, length of stay; RevKR, revision total knee replacement.

**Figure 5:**
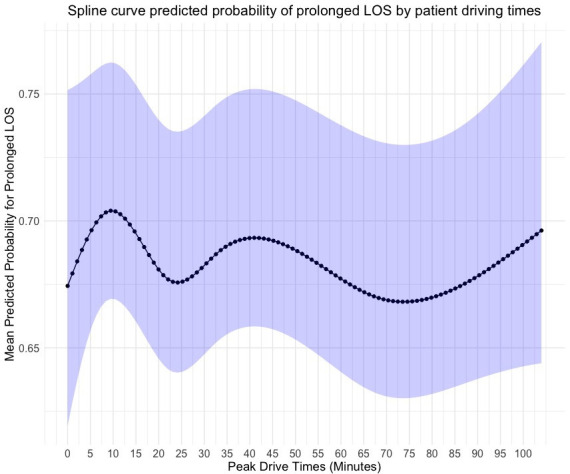
Predicted probability of prolonged length of inpatient stay by patient driving time from hospital after RevKR. A fixed effects multivariable logistic regression model using 4 knots at 5%, 35%, 65% and 95% centiles of mean unit volume. 95% CIs represented by blue shaded line. LOS, length of stay; RevKR, revision total knee replacement.

## Discussion

### Statement of principal findings

We present a multihospital site retrospective analysis of patients undergoing revision knee replacement surgery at tertiary referral centres in England. In this analysis of 6880 patients undergoing RevKR, we did not observe a statistical association between distance and time travelled for revision surgery and readmission within 30 days.

### Strengths and weaknesses of the study

The findings of this study should be interpreted in view of several limitations. First, this analysis used observational data from a large administrative dataset covering all NHS-funded procedures conducted in England. As with all administrative datasets, we are limited in the amount of detail provided regarding presentation. We chose to categorise a high-volume centre by trust to accurately capture surgical experience. All NHS hospitals in England are run by hospital trusts which typically involve between one and four hospitals within a catchment area standardising their practice. It is common practice for specialist orthopaedic surgeons to move between these sites delivering the same procedures. Our study involved 187 hospital sites run by 38 trusts. We acknowledge this is a weakness of our study as this may not be representative of all trusts. We included all indications for RevKR in our patient cohort because indication was not thought to be related to how far a patient lives from a hospital. However, we acknowledge the rate of complications is higher in patients with infection, and we subsequently adjusted for indication for revision in our analyses.^26^ It is likely that because we did not exclude previous revision knee arthroplasty patients, the complexity of the surgery undertaken in our cohort varied. We recognise this is a limitation of the study; however, we assume casemix was unrelated to patient travel distance.

There were many missing patients (approximately 36%) following the linkage of HES data with Journey Time Statistics. To account for this, we assumed that the data were missing at random and used multiple imputation to estimate missing travel distances. It is likely the imputed values may introduce bias; however, we modelled these based on predictors and dependent variables to improve our estimates. We do not present a sample size calculation; rather, we have used all available data, and our sample size was set by our inclusion criteria. We controlled for the clustered nature of our data between hospital providers through inclusion as a covariate in our modelling. To ensure consistency in our definition of tertiary referral hospitals, only hospitals performing >49 revisions/year were included. These are likely to treat a similar casemix of patients and potentially have similar access to resources within a national healthcare system. This approach allowed us to control for variation across providers. However, we acknowledge it does not fully account for the hierarchical nature of the data with differences in treatment protocols and hospital specialisation among factors which may influence patient outcomes.

There is a lack of granular clinical data using HES for each readmission. Therefore, we cannot ascertain precise reasons for readmissions, but we assume they are related to a postsurgical complication. Information on the exact date of readmission and death was also not available. Therefore, a time-to-event approach in outcome analysis was not possible. Clinical coding practice within HES is known to vary across trusts.^27^ As an example, some trusts may be more consistent in coding comorbidities, and this may have created some bias. However, this is unlikely to vary systematically with travel distances and so significantly bias our findings. We acknowledge the relatively short travel distances in this population compared with examples from the USA. As such, the results of this study may not be generalisable to larger geographical areas or less mature healthcare systems. However, the upper quintile in our study represents a substantial journey distance and time for our patient cohort, where poor mobility is a significant factor affecting their care. This analysis does not consider journey times of those who may not have access to a car and instead chose to take public transport.

### Strengths and weaknesses in relation to other studies, discussing important differences in results

This is the first study to analyse the potential impact of patient travel distances on patients receiving RevKR. The findings that longer travel distances are not associated with inferior outcomes are an important part of the evaluation of the assumptions and context behind the establishment of revision knee networks.^28^ This study has shown that concerns about introducing a network in larger geographical regions, for example, in Scotland, where longer patient travel distances and times are common, may be less important.^29^ This is particularly useful as regions explore the geography of their revision networks and during summative outcome assessment of this complex health intervention.^30^ Despite there being a potential negative association between straight line travel distance and emergency readmission at 30 days, there was a lack of association involving driving distances and times which present real world challenges for patients.

It may be seen as surprising that no association between travel distance and prolonged length of hospital stay was identified. An expectation exists of increasing difficulties being encountered with the discharge of patients living greater distances from their treating hospital, which has been observed in patients following elective pancreatic surgery.^31^ This is also an observation seen in patients being treated in specialist vascular centres in the USA, which led to the recommendation of additional care coordination and follow-up efforts. However, the geography of the population in these studies was much larger with significantly longer travel distances.

We did observe a weak but statistically significant correlation between social deprivation status and age of the patient with longer travel distances. Patients from poorer sociodemographic backgrounds may be expected to travel further for RevKR. This highlights the additional care coordination and follow-up efforts that should accompany the widening reach of regional revision knee networks. It is reassuring that access to treatment for older patients is unaffected by travel distance. However, there may be patients who refused to travel to a specialist centre and opted for treatment at their local centre.

### Meaning of the study: possible explanations and implications for clinicians and policy-makers

The organisation and delivery of revision knee services in England has recently undergone a substantial change, and now such services are provided around regional networks of care. This promises substantial advantages to the increasing number of patients with problematic knee replacements in our ageing population who will benefit from regional expertise.^8^ However, it is unknown the impact of patients residing farther from tertiary referral centres, particularly rural patients who may encounter additional difficulties associated with greater travel distance. A recent study following the outcomes of aortic surgery found that longer travel distances are associated with inferior perioperative outcomes.^13^ Similar associations have been found in postoperative colorectal surgery patients.^32^ As such, our results are reassuring to policy makers and clinicians.

### Unanswered questions and future research

There is a scarcity of evidence evaluating the patient perception of complex health interventions such as network models of care. Recent work by Kugler *et al* has demonstrated the willingness of patients to travel further for better outcomes in the context of total knee replacement surgery.^33^ Nevertheless, patient perceptions of travelling further for their treatment should be a focus for future research in the context of revision knee patients, particularly as this is 1 of the top 10 research priorities identified by the James Lind Alliance priority setting partnership.^34^

## Conclusions

We did not observe an association in our study population between 30-day readmission rates and increasing travel distances or times between a patient’s home and their treating hospital in revision knee replacement. This paper is the first to explore the relationship between travel distance and complex orthopaedic surgery and informs some concerns regarding the creation of a centralised revision knee network. This information is of utility to surgical providers and commissioners of healthcare services. Furthermore, it can inform patient-led decision-making and the exploration of perceptions surrounding travelling for complex surgery. Although this is the first assessment in complex orthopaedic surgery, a prospective analysis will be undertaken as part of the ongoing auditing of revision knee networks in England.

## Supplementary material

10.1136/bmjopen-2024-085201 online supplemental file 1

10.1136/bmjopen-2024-085201 online supplemental file 2

10.1136/bmjopen-2024-085201 online supplemental file 3

10.1136/bmjopen-2024-085201 online supplemental file 4

10.1136/bmjopen-2024-085201 online supplemental file 5

## Data Availability

All data relevant to the study are included in the article or uploaded as supplementary information.
