# Supplementary material for: What is the impact of longer patient travel distances and times on perioperative outcomes following revision knee replacement: a retrospective observational study using data for England from Hospital Episode Statistics
Source: BMJ Open. 2025 May 6;15(5):e085201. doi: 10.1136/bmjopen-2024-085201 (PMC12056618; doi:10.1136/bmjopen-2024-085201)
Supplement: online supplemental file 1 [file bmjopen-15-5-s001.pdf]

# Journey Time Statistics: Notes and Definitions

## About this release

This publication supports the latest statistics on journey times.

## In this publication

Overview......................p1
Access to key services ....................................p4
Connectivity..................p7
Data sources ...............p9
Outputs ......................p18
Strengths and weaknesses ....................................p19

## Further information

**Public enquiries**
020 7944 3077
vehicles.stats@dft.gov.uk
**Media enquiries**
020 7944 3066

## Overview

This note provides information on the methodology used, the source data and definitions of key terms for calculating Journey Time Statistics.

These annual statistics were first published in December 2015 for the year 2014 and have been developed from the earlier Accessibility Statistics published for 2007 to 2013.

The Journey Time Statistics produced by DfT consists of theoretical journey times calculated by modelling journeys between known sets of origins and destinations. It uses information on the road network, traffic speeds and public transport timetables in England.

The relevant Journey Time Statistics calculation is varied for origins and destination to meet a variety of needs. Two sets of analysis are published:

- ► Access to key services; and
- ► Connectivity

### Origin indicators

These indicators measure the number of different services in a particular area that users can reach within a given time.

### Destination indicators

These indicators measure the proportion of users that can access a service within a certain time.

The 'user' populations for each service in the destination indicators are:

| | |
|---|---|
| Employment | 16-74 year olds |
| Primary schools | 5-10 year olds |
| Secondary schools | 11-15 year olds |
| Further education | 16-19 year olds |
| All other services | All households |

## Key services

- Employment centres: Data used are the number of jobs in a Lower Super Output Area (LSOA). The data tables include results for employment centres of 3 different sizes (100-499 jobs, 500-4,999 jobs and at least 5,000 jobs). For the key services average, the 500-4,999 jobs definition is used for employment.
- Education: Locations of all open Primary schools, Secondary schools, Further Education and Sixth Form Colleges.
- General Practice (GP) surgeries: For 2017 based on the Patients Registered at a GP Practice dataset released by NHS Digital – previously this was based on a filtered dataset of NHS prescribers released by NHS Digital.
- Hospitals: Based on hospitals that are registered with the Care Quality Commission (CQC) and are managed by Acute Trusts.
- Food stores: Locations of grocery, supermarkets or convenience stores.
- Town centres: Locations of Town centres using a central focal point for the town mapped to the nearest road.

## Geography

- Local authorities

In some parts of England there are two tiers of local authorities, and in others a single unitary authority. Statistics have been calculated for both types of authority - around 360 in all. These vary considerably in size, from a population of a few tens of thousands to over a million.

- Lower Layer Super Output Areas (LSOA)

LSOAs are small areas designed to be of a similar population size, with an average of approximately 1,500 residents or 650 households. There are 32,844 Lower-layer Super Output Areas (LSOAs) in England. They were determined by the Office for National Statistics for the reporting of small area statistics and are derived from the 2011 Census.

- Urban and rural definitions

This report uses the Defra Rural-Urban Classification, based on 2011 Census Output Areas. The Rural-Urban Classification defines areas as rural if they fall outside of settlements with more than 10,000 resident population. See Defra's Definitions and Local Authority Classification for more details.

## Journey time calculations

The journey time calculations are carried out using a commercially available software package called TRACC, owned by Basemap. TRACC is a desktop application that uses public transport and highways data to create journey times from origins to destinations. It uses timetable information showing both arrival and departure times at stops from public transport services against a specific time/day period. Highways information from road networks are used to fill the gaps between public transport services by creating a linear network that connects the origins, destinations and stops together. This provides a fully routable network of nodes and lines which is saved on file as a graph network. The graph network has various constraints which can be altered to suit the user need such as distance travelled, interchange delays on public transport and stopping limitations on road networks. The TRACC software then queries the graph network with origin and destination co-ordinates and uses the Dijkstra shortest path algorithm to route between these points. This is an algorithm for finding the shortest distance for travel between the graph networks.

For a public transport journey, the journey time produced includes all walking elements of the journey, i.e. the walk from the origin of the journey to the road, from the road to public transport stops, any interchange of public transport using the road and then from the final stop to the destination via the road, and finally from the nearest point on the road network to the destination. The journey assumes arrival at the first stop one minute before the initial departure, with any subsequent interchange waiting times included as part of the final journey time.

Car, cycle or walk only journeys are similar except that once the road network is reached the journey proceeds link by link along the road network at speeds governed by data held in the model. These are specific to the mode, the road type, and in some cases the individual road link.

The 10 shortest journey times from each origin (i.e. Output Area) are calculated for each destination type. For the public transport / walking mode these consist of the 10 shortest journey times by either walking or public transport, after applying a 5 minute penalty for any journeys using public transport (to represent travellers arriving slightly early at the first stop).

The journey times are representative of the ‘morning peak’. This is made explicit for public transport / walking by requiring the journey to be completed between 7 and 10am, and for car journeys by using average traffic speeds for between 7 and 10am. For the cycle mode no actual speed data are available. The cycle speeds used are default assumptions, and are not based on a particular time of day.

## Access to key services

The Access to Services analysis applies the Journey Times methodology to origins consisting of residential neighbourhoods and destinations consisting of centres of employment and a range of key local services. Journey times are calculated for three modes of transport: public transport; driving; and cycling. These journey times are then used to generate further indicators, as described in the **Outputs Section**.

The Access to Services calculation process and the coverage of the data set are very similar to those of the Accessibility Statistics from which they were developed. However, the calculation algorithm and a number of other features of the design are different, so the results are not directly comparable.

The statistics are designed to represent as much as possible the situation on a **Tuesday in October** of **the year to which they relate**. Data for the second week of October are used in the analysis, since this provides a fairly typical week, unaffected by major national holidays, school holidays or other seasonal effects. The origins, destinations and public transport timetables used are as far as possible for this date. The traffic data are averages for the preceding 12 months up to and including August. The road networks are those current at the start of the traffic data year.

### Outline of access to services calculation process

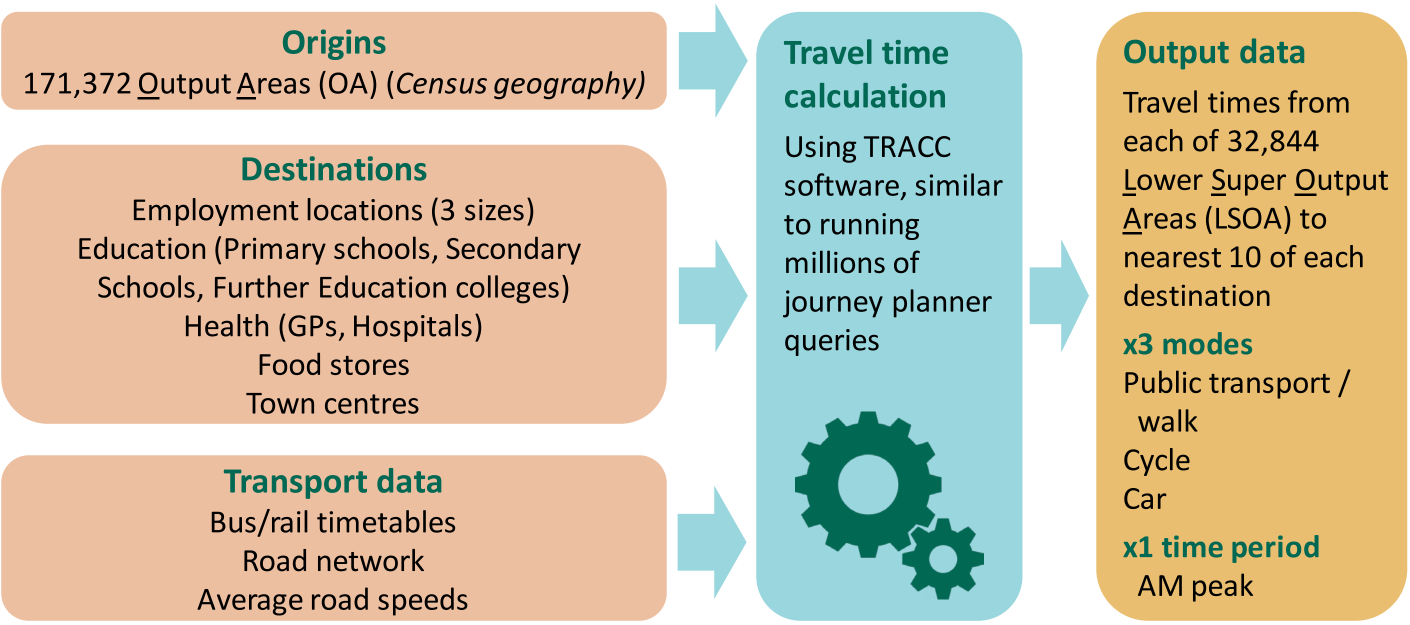


# Model parameters and assumptions

## General parameters

Maximum journey time of **2 hours.**

Maximum journey distance of **100km.**

## Walking

These apply to both:

► walking between origin / destination and the transport networks at both ends of a journey by

any mode;

► walk only journeys as part of the public transport / walk mode.

Maximum straight line distance between origin / destination and road network of **2km.** The algorithm will always use nearest point on network. For cycle or car modes, travel by cycle or car begins from this point. For public transport/walk, traveller walks along road network to the most suitable public transport stop, or direct to the destination if this is quicker.

Walking speed on road/path network of **4.8km/h.**

Walking speed off road/path network of **4.0km/h.**

## Public transport

Interval within which door-to-door journey must be completed (required for timetable selection) is **7am to 10am on a Tuesday.**

Maximum walk distance of **3km** - this applies to walks from origin to first public transport stop, from last stop to destination, and also walking directly from origin to destination without using public transport at all.

Maximum number of potential first public transport stops considered in routing algorithm is **100** (starting with the closest to origin).

Allowance for catching first public transport service is **5 minutes** - added to any journey that involves boarding one or more public transport services.

Public transport speed – this is provided implicitly by the timetable information.

Interchange time of **5 minutes** (minimum interval allowed between arriving at a stop and catching another service).

Maximum straight line distance between public transport interchanges of **500m**.

Stop clustering at **150m** – groups together public transport stops within this distance of one another to speed up processing. The individual timetables for each service are retained.

## Cycling speeds

| Road Type | Speed |
|---|---:|
| Motorway | 0.0 km/h |
| Urban Motorway | 0.0 km/h |
| A road | 16.0 km/h |
| B road | 16.0 km/h |
| Minor road | 16.0 km/h |
| Local street | 16.0 km/h |
| Private road – restricted access | 4.8 km/h |
| Private road – public access | 16.0 km/h |
| Pedestrian street | 4.8 km/h |
| Alley | 4.8 km/h |

Parking time of **5 minutes** - added to all cycle journeys.

## Car speeds

| Type of road | 2014 | 2015 | 2016 | 2017 |
|---|---|---|---|---|
| | Default speeds (km/h) | | | |
| Motorway | 79.5 | 77.0 | 77.5 | 77.6 |
| Urban Motorway | 79.5 | 77.0 | 77.5 | 77.6 |
| A road | 42.7 | 43.7 | 43.3 | 43.2 |
| B road | 41.6 | 43.0 | 42.2 | 41.9 |
| Minor road | 36.8 | 37.5 | 36.8 | 36.3 |
| Local street | 19.2 | 17.8 | 18.8 | 18.3 |
| Private road – restricted access | 17.0 | 16.7 | 16.2 | 15.3 |
| Private road – public access | 14.8 | 15.2 | 15.1 | 13.6 |
| Pedestrian street | 0.0 | 0.0 | 0.0 | 0.0 |
| Alley | 0.0 | 0.0 | 0.0 | 0.0 |

Car speeds are calculated for specific links where more than 200 records exist otherwise the default speeds are used. Minimum journey time for a journey that uses a car is **5 minutes**.

## Time at junctions

Road normalisation is used for all modes of transport which converts each road link to a straight line to speed up processing. The true link length is retained for accurate speed/time calculations, but there could be a small effect on the calculation of shortest distance from the road network to destination points. Effect for origins is minimal due to origins being constrained to road nodes.

## Connectivity

These experimental analyses are intended to apply the Journey Times methodology to a range of more strategic or economically significant destinations than the primarily local services covered by the Access to Services analyses; including airports and railway stations. The principle difference in the Connectivity approach from that of the Access to Services analyses is that journey times are calculated, as far as possible, to all accessible locations, rather than to just the nearest 10 examples. This tends to result in a much larger data set being generated. In some cases a longer maximum journey time may be allowed although this may depend on what is considered reasonable for the type of destination. Given these factors, a less detailed origin data set may be used than for Access to Services. This is both necessary, to limit the size of the data set, and acceptable where the typical journey lengths are longer.

The first connectivity analyses published using the new Journey Time methods were released in Journey Time Statistics 2015, published in April 2017, for two destination sets – airports and rail stations. These analyses using the Journey Times methods superseded two earlier Connectivity Statistics reports published in 2014 and 2015 based on the old accessibility statistics methods, in the same way that the new Access to Services analyses have replaced the earlier Accessibility Statistics. Again, the connectivity results produced using the old and new methods are not directly comparable.

### Outline of Connectivity calculation

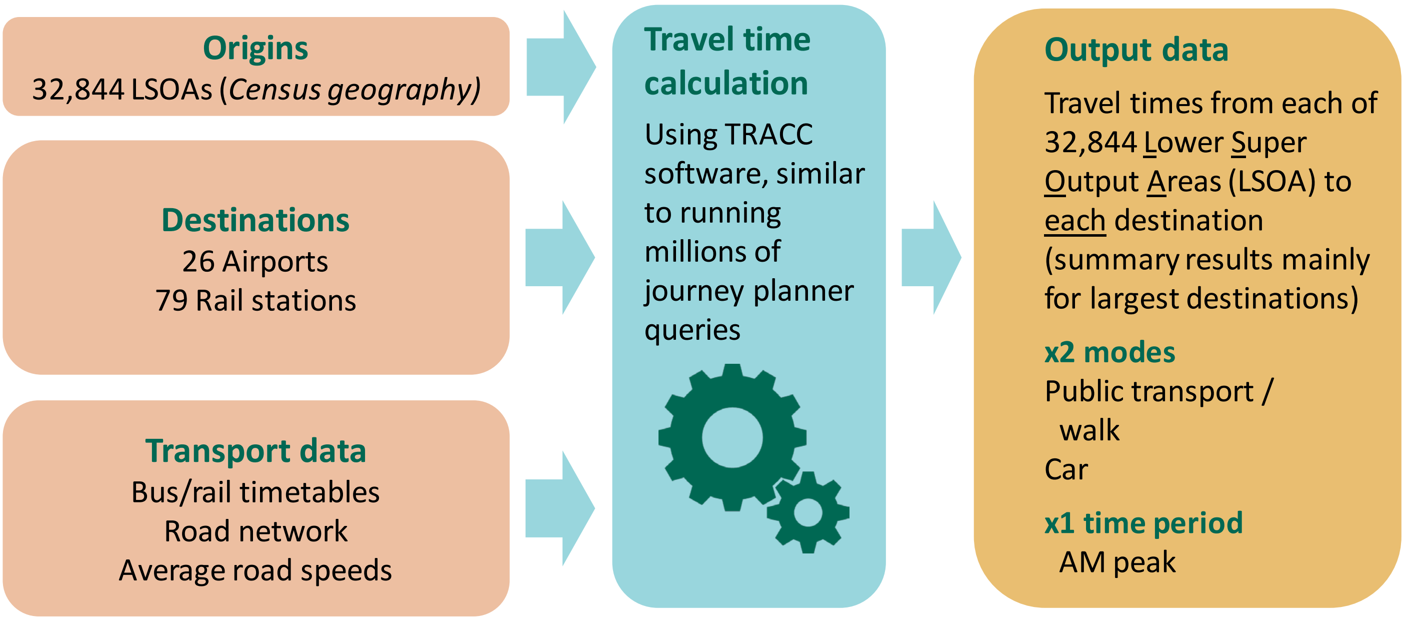


### Model parameters and assumptions

| | |
|---|---|
| Origins | Population weighted centroids (the central point) of 32,844 English LSOAs as specified in the 2011 Census geography. These points were then constrained to the nearest road node, as for Access to Services method. |

| | |
|---|---|
| Journey Time Calculation | As for Access to Services, for public transport / walking and car modes only, except that a maximum journey time of 240 minutes and maximum straight line distance of 400km is allowed. |
| Outputs | Generally similar to Access to Services, with different journey time classifications as appropriate. Journey time results to specific destinations are included – this is the key difference in the Connectivity analyses. ‘Average journey times’ and ‘nearest’ destinations should be used with caution. The average journey times exclude results for areas with no available connection under 240 minutes, which may become significant in remote areas and for destinations are a great distance from the origin. The ‘nearest’ destination is the destination with the shortest average journey time across the whole area considered – which will be relatively large in the case of local authority level results. |

# Data sources

## Origins

The origins used for all Access to Services calculations are the 171,372 English Output Areas (OA) as specified in the 2011 Census geography.

To provide the actual journey start point in each OA, the population weighted centroid of the OA was shifted to the nearest node (i.e. junction) on the road network. This was to avoid biasing the journey time results where the centroid of the OA was a long way from a road. In fact it is rare for an OA centroid to be more than about 100 metres from a road – only a tiny handful of OA in remote areas have centroids as much as 1km from a road. The OA centroids have been shifted onto the nearest road node rather than the nearest point on a road in order to reduce issues arising from normalising the road network.

| Origin | Data source for the origin points |
|---|---|
| All | Data: Population centroid of each Output Area in 2011.<br><br>Source: ONS 2011 Census Boundaries.<br><br>Further information: http://geoportal.statistics.gov.uk |

## Destinations

The destinations used consist of three different sizes of employment centre and the locations of seven other types of key local service. For each of these key services a nationally consistent data set has been identified or derived – further information on these is provided in this section.

Each destination is located by a 6-figure National Grid reference. For the employment destinations this is taken to be the population weighted centroid of the LSOA.

| Destination | Number of locations | | | |
|---|---|---|---|---|
| | 2014 | 2015 | 2016 | 2017 |
| Employment centres (small) | 16,465 | 16,625 | 16,930 | 17,194 |
| Employment centres (medium) | 9,235 | 9,460 | 9,707 | 10,241 |
| Employment centres (large) | 645 | 676 | 719 | 785 |
| Primary schools | 16,463 | 16,484 | 16,655 | 16,927 |
| Secondary schools | 3,365 | 3,376 | 3,381 | 3,174 |
| Further education colleges | 2,624 | 2,606 | 2,418 | 2,304 |
| GPs | 9,257 | 11,167 | 9,128 | 7,353 |
| Hospitals | 296 | 278 | 278 | 277 |
| Food stores | 19,549 | 19,746 | 21,665 | 20,987 |
| Town centres | 1,211 | 1,211 | 1,211 | 1,211 |

The data source for GP surgeries was reviewed and replaced for 2017.

## Access to key services

| Destinations 2017 | Data source for the locations of the service | Data source for users of the service |
|---|---|---|
| Employment | Data: Number of jobs available in a LSOA in the year before the calculation year. | Data: Number of 16-74 year olds in each output area. |
| | Source: ONS Business Register Employment Survey. | Source: ONS mid-year population estimates for calculation year. |
| | Further information: https://www.nomisweb.co.uk/default.asp | Further information: ONS mid-year population estimates: http://www.ons.gov.uk/ons/taxonomy/index. |
| Primary schools | Data: Location of all open primary schools in September of calculation year. | Data: Number of 5-10 year olds in each output area. |
| | Source: The Department for Education (DfE) Edubase. | Source: ONS mid-year population estimates for calculation year. |
| | Further information: https://get-information-schools.service.gov.uk/ | Further information: ONS mid-year population estimates: http://www.ons.gov.uk/ons/taxonomy/index. |
| Secondary schools | Data: Location of all open secondary schools in September of calculation year. | Data: Number of 11-15 year olds in schools in each output area. |
| | Source: DfE Edubase. | Source: ONS mid-year population estimates for calculation year. |
| | Further information: https://get-information-schools.service.gov.uk/ | Further information: ONS mid-year population estimates: http://www.ons.gov.uk/ons/taxonomy/index. |
| Further education colleges | Data: Location of all open further education and sixth form colleges/school sixth form in September of calculation year. | Data: Number of 16-19 year olds in each output area. |
| | Source: DfE Edubase. | Source: ONS mid-year population estimates for calculation year. |
| | Further information: https://get-information-schools.service.gov.uk/ | Further information: ONS mid-year population estimates: http://www.ons.gov.uk/ons/taxonomy/index. |

| Destinations 2017 | Data source for the locations of the service | Data source for users of the service |
|---|---|---|
| GPs | Data: Locations of GP surgeries with registered patients in October of calculation year. | Data: Number of households in each output area. |
| | Source: NHS Digital table of Registered patients at GP practices | Source: 2011 Census + Local Authority (LA) updates from the Ministry of Housing, Communities & Local Government (MHCLG) mid-year household projections of calculation year. |
| | Further information: https://digital.nhs.uk/data-and-information/publications/statistical/patients-registered-at-a-gp-practice | Further information: 2011 Census: http://www.nomisweb.co.uk/census/2011<br>MHCLG mid-year household projections: https://www.gov.uk/government/statistical-data-sets/live-tables-on-household-projections |
| Hospitals | Data: Location of hospitals. | Data: Number of households in each output area. |
| | Source: Care Quality Commission - Directory of places that provide care. | Source: 2011 Census + LA updates from MHCLG mid-year household projections of calculation year. |
| | Further information: http://www.cqc.org.uk/content/how-get-and-re-use-cqc-information-and-data | Further information: 2011 Census: http://www.nomisweb.co.uk/census/2011<br>MHCLG mid-year household projections: https://www.gov.uk/government/statistical-data-sets/live-tables-on-household-projections |

| Destinations 2017 | Data source for the locations of the service | Data source for users of the service |
|---|---|---|
| Food stores | Data: Location of grocery/ supermarkets or convenience stores in October of calculation year. | Data: Number of households in each output area. |
| | Source: The Local Data Company | Source: 2011 Census + LA updates from MHCLG mid-year household projections of calculation year. |
| | Further information: https://www.localdatacompany.com/ | Further information: 2011 Census: http://www.nomisweb.co.uk/census/2011<br>MHCLG mid-year household projections: https://www.gov.uk/government/statistical-data-sets/live-tables-on-household-projections |
| Town centres | Data: Location of town centres in 2004. | Data: Number of households in each output area. |
| | Source: MHCLG Town Centre and retail planning statistics for England and Wales. | Source: 2011 Census + LA updates from MHCLG mid-year household projections of calculation year. |
| | Further information: https://data.gov.uk/dataset/ed07b21f-0a33-49e2-9578-83ccbc6a20db/english-town-centres-2004 | Further information: 2011 Census: http://www.nomisweb.co.uk/census/2011<br>MHCLG mid-year household projections: https://www.gov.uk/government/statistical-data-sets/live-tables-on-household-projections |

## GP destination data

The GP surgery destinations used from 2014 to 2016 are based on the list of practices maintained by the Organisational Data Service of the Health & Social Care Information Centre, and published at https://digital.nhs.uk/services/organisation-data-service/data-downloads/gp-and-gp-practice-related-data. This was supplemented with information on branch surgeries from the same source. Grid references were derived from the postcode using the Office for National Statistics (ONS) Postcode Address File. Practices with identical postcodes were taken to be duplicates or co-located, and all additional records after the first were removed.

From 2017, the list of GP locations is taken from the NHS Digital publication of Registered patients at GP practices for October of the calculation year. This had the effect of reducing the number of locations in the dataset, but removed the need for manual adjustments and produces a more stable list defined as GP practices with registered patients. Grid references were derived from the postcode using the Office for National Statistics (ONS) Postcode Address File.

## Hospital destination data

The starting point for hospital sites is the Care Quality Commission's (CQC) list of 'active locations' dataset, which is thought to be the most-up-to date and freely available source of data on individual National Health Service (NHS) and social care 'sites' or hospitals. A criteria was developed in consultation with the Department of Health to reduce the list down to capture only the key hospitals. The following have been removed and individual records have been inspected to remove further examples of these cases and for any duplicates:

- care home records;
- non-NHS providers;
- sites not associated with acute providers;
- any remaining sites that are associated with Specialist Trusts (usually single speciality Trusts or Sites);
- records where it is evident from the name that the record is not a hospital (e.g. headquarters, specialist units.)

This gave a final list of 278 hospitals in 2017 run by Acute (non-specialist) Trusts. As well as covering all general hospitals this will still include some with a largely or entirely community or rehabilitation role, where these happen to be managed by an Acute Trust. It was considered on balance better to leave these in the list, rather than risk adding further subjectivity to the selection. Whilst not perfect, it is considered that the resulting list is a significant improvement on that used previously.

| **Steps taken to produce hospital data set** |
| --- |
| Remove records where **Care Home** = Y |
| Remove records where **Provider ID** begins 1- |
| Keep records where **Benchmark Group** is Care Home or **Cluster Group** is Acute |
| Filter the trust site locations by name to remove obvious non-hospital sites. Key words used for this process are: birth, dental, house, clinic, grange, lodge, infirmary, health, community, unit, surgery, centre |
| Manual review of remaining locations |

## Employment destination data

The employment centres are defined by the number of jobs existing in each English LSOA, taken from the Business Register Employment Survey. Large Employment Centres are defined as those with 5,000 or more jobs, Medium Employment Centres as those with 500 or more jobs, up to 4,999 and Small Employment Centres as those with 100 or more jobs, up to 499.

Data are downloaded from the Nomis website; although LSOA level BRES data has safeguarded access, access can be requested through the site. The chosen data download options are LSOA2011 geography, date as calculation year, variable as employment status where the value is employed, and the measure chosen is a count.

For the 2016 destination set, the BRES changed from 2001 census geography to 2011 census geography. The majority of LSOA boundaries are unchanged between these datasets, but some have been merged or split. Therefore the employment destination indicators are not strictly comparable between 2015 and 2016 Journey Time statistics. See https://www.ons.gov.uk/methodology/geography/ukgeographies/censusgeography for further information.

## Education destination data

The education destination datasets are taken from the Department for Education database of educational establishments. The database was filtered to remove those establishments that were not open during the school year starting in September of the calculation year. Further filters were applied to remove special educational establishments, boarding schools and selective schools, and then to select schools at each phase of education for primary and secondary schools and further educational establishments. The following table lists the filters used.

| Phase of Education | Code Variable | Variable | Selected codes and values | |
|---|---|---|---|---|
| All Schools | OpenDate | | | 30/08/17 or earlier; NULL |
| | CloseDate | | | 30/08/18 or later; NULL |
| | TypeOfEstablishment_Code_ | TypeOfEstablishment | 1 | Community school |
| | | | 2 | Voluntary aided school |
| | | | 3 | Voluntary controlled school |
| | | | 5 | Foundation school |
| | | | 6 | City technology college |
| | | | 12 | Foundation special school |
| | | | 18 | Further education |
| | | | 28 | Academy sponsor led |
| | | | 29 | Higher education institutions |
| | | | 31 | Sixth form centres |
| | | | 32 | Special post 16 institution |
| | | | 34 | Academy converter |
| | | | 35 | Free schools |
| | | | 36 | Free schools special |
| | | | 39 | Free schools 16 to 19 |
| | | | 40 | University technical college |
| | | | 41 | Studio schools |
| | | | 45 | Academy 16-19 converter |
| | | | 46 | Academy 16 to 19 sponsor led |
| | Boarders_Code_ | Boarders | 0 | Not applicable |
| | | | 1 | No boarders |
| | | | 9 | NULL |
| | AdmissionsPolicy_Code_ | AdmissionsPolicy | 0 | Not applicable |
| | | | 4 | Non-selective |
| | | | 9 | NULL |
| Primary schools | PhaseOfEducation_Code_ | PhaseOfEducation | 2 | Primary |
| | | | 3 | Middle deemed primary |
| | | | 7 | All through |

| Phase of Education | Code Variable | Variable | Selected codes and values | |
|---|---|---|---|---|
| Secondary schools | PhaseOfEducation_Code_ | PhaseOfEducation | 0 | Not applicable |
| | | | 4 | Secondary |
| | | | 5 | Middle deemed secondary |
| | | | 7 | All through |
| | Statutory High age | | >=16 | |
| | Statutory Low age | | < 16 | |
| FE | PhaseOfEducation_Code_ | PhaseOfEducation | 4 | Secondary |
| | | | 5 | Middle deemed secondary |
| | | | 6 | 16 plus |
| | | | 7 | All through |
| | Statutory High age | | >16 | |
| | OfficialSixthForm_Code_ | OfficialSixthForm | 0 | Not applicable |
| | | | 1 | Has a sixth form |
| | | | 9 | NULL |
| **OR** | | | | |
| FE | EstablishmentTypeGroup__code_ | EstablishmentTypeGroup | 1 | Colleges |

## Food Stores destination data

The food stores destination dataset is purchased from The Local Data Company and includes all branches of multiple food store chains. Although some data are available for independent food stores, this only exists within town centres and so has not been included.

## Connectivity

| Destinations | Data source for the locations of the service | Data source for users of the service |
|---|---|---|
| Airports | Data: Location of GB airports excluding highlands and islands of Scotland<br><br>Source: National Public Transport Access Nodes<br><br>Further information: https://data.gov.uk/dataset/ff93ffc1-6656-47d8-9155-85ea0b8f2251/national-public-transport-access-nodes-naptan | Data: Number of households in each output area.<br><br>Source: 2011 Census + LA updates from MHCLG mid-year household projections of calculation year.<br><br>Further information: 2011 Census: http://www.nomisweb.co.uk/census/2011<br><br>MHCLG mid-year household projections: https://www.gov.uk/government/statistical-data-sets/live-tables-on-household-projections |

| Destinations | Data source for the locations of the service | Data source for users of the service |
|---|---|---|
| Railway stations | Data: Location of larger (category A, B and C1) rail stations in GB<br><br>Source: Network rail classification<br><br>Further information:<br><br>http://webarchive.nationalarchives.gov.uk/20101007153226/http://www.dft.gov.uk/pgr/rail/passenger/stations/betterrailstations/<br><br>http://archive.nr.co.uk/browse%20documents/rus%20documents/route%20utilisation%20strategies/network/working%20group%202%20-%20stations/networkrusstations.pdf | Data: Number of households in each output area.<br><br>Source: 2011 Census + LA updates from MHCLG mid-year household projections of calculation year.<br><br>Further information: 2011 Census: http://www.nomisweb.co.uk/census/2011<br><br>MHCLG mid-year household projections: https://www.gov.uk/government/statistical-data-sets/live-tables-on-household-projections |

## Transport network data

Travellers moved between their original and their destination via one or more of the following transport networks, depending on the mode of transport being modelled. For all modes, travellers will probably also need to walk between their origin / destination and the transport network. For some short journeys, it may be quicker for travellers to walk directly to their destination, rather than using public transport at all – this is why public transport / walking results are modelled as a combined mode.

### Public transport

National public transport timetable data are publically available. Data for bus, local coach and other local transport services (e.g. light rail, metro, and ferry) are captured in the Traveline National Data Set (TNDS), rail timetable data are published by the Association of Train Operating Companies (ATOC), and national coach services in the National Coach Data Set (NCDS).

### Walk

The walking network is represented by the road and urban path elements of the Integrated Transport Network produced by the Ordnance Survey.

### Cycle

The cycling network is represented by the road network including cycle paths and bridleways from the Integrated Transport Network. Cycle journeys are also allowed to use footpaths at walking pace.

## Car

The car network is represented by the road component of the Integrated Transport Network.

Data on actual vehicle speeds on each road network link (generally the stretch of road between 2 nodes, or junctions) is obtained from Trafficmaster Satnav devices and are used to estimate car speeds. These data are used to calculate annual average traffic speeds on each link of the road network (by direction if the link is bi-directional). These are used as the link speeds for cars in the modelling. Where the Trafficmaster sample for an individual link is too small, national averages of the same data for the particular road type are used instead. This is an innovation from 2014. Previously the sample was too small and the model reverted to default assumptions for car speed based on road type which were much higher than the Trafficmaster averages, resulting in some inconsistency in the model.

# Outputs

The journey time results are used to create the following indicators for publication:

| Indicator | Description |
|---|---|
| Minimum journey time | The shortest of the ten journey time results. |
| Origin indicators | Four measures, the number of destinations (up to the maximum of 10) that can be reached from a given origin within 15, 30, 45 and 60 minutes. |
| Destination indicators | Four measures, the percentages of service users within the given geographical area who can access at least one service location within 15, 30, 45 and 60 minutes. |

Each of these indicators is calculated for each mode and each destination type, and at a number of geographical scales as follows:

- England
- Region
- Local Authorities, including London Boroughs, Metropolitan districts, Unitary authorities, Counties and non-Metropolitan districts, also Inner and Outer London and former Metropolitan counties
- 2011 Lower layer Super Output Area
- 2011 Defra Rural/Urban Classification

The indicators for each geography are calculated as population weighted averages. In other words, the average minimum journey time for an area, B, is:

$$\mathbf{mjt(B)= \sum_{i=1}^{n}(mjt(OA_i)\times pop(OA_i))/pop(B)}$$

where mjt(B) is the minimum journey time in area B, mjt(OAi) is the minimum journey time of the ith of n output areas making up area B, and pop(B) and pop(OAi) are the user populations resident in area B and output area i respectively.

The service user populations used in the above weighting, and in the destination indicators, depend on the destination type, as follows:

| Destination type | Service user population basis |
|---|---|
| Employment centres | Resident population of working age (16-74 years) |
| Primary schools | Population aged 5-10 |
| Secondary schools | Population aged 11-15 |
| Further education colleges | Population aged 16-19 |
| GPs, hospitals, food stores, town centres | Number of households |
| Average key services | Resident population of working age (16-74 years) |

## Strengths and Weaknesses

In using the data, the following points should be kept in mind:

► All journey times are compiled on a consistent basis across the country.

► The statistics are based on the calculation of theoretical journey times, they are not based on real journeys. They are however based on actual public transport times, and average traffic speeds on the road network.

► Although the statistics are calculated to a high level of geographical detail, some assumptions and simplifications are necessary in the modelling (for example assigning the start point of journeys to a single point in each Output Area, road speeds, interchange times for public transport).

► For 2016 we have used the 2015 BRES data to designate Lower Super Output Areas as employment centres. The 2015 BRES is the first year to use LSOAs based on the 2011 census, and although the majority of these are an exact match to the 2001 LSOAs, there are some that were merged, split or had other boundary changes. For these areas journey times from earlier years are not comparable to the 2016 journey times. This effect is more pronounced for large employment centres, as there are fewer destinations to route to.

► For particular areas, local authorities and other experts may have more detailed information allowing them to produce more accurate or detailed models of the local situation.

► Demand responsive services (e.g. bus services which have to be booked) are only included to the extent that they can be plausibly modelled, in the Traveline National Data Set.

► Since new journey calculation software was adopted for 2014, along with a significant number of other changes to the methodology, from 2014 results are not directly comparable with those for earlier years.
