# Supplementary material for: What is the impact of longer patient travel distances and times on perioperative outcomes following revision knee replacement: a retrospective observational study using data for England from Hospital Episode Statistics
Source: BMJ Open. 2025 May 6;15(5):e085201. doi: 10.1136/bmjopen-2024-085201 (PMC12056618; doi:10.1136/bmjopen-2024-085201)
Supplement: online supplemental file 2 [file bmjopen-15-5-s002.docx]

## Supplementary material S1 – OPCS-4 code criteria used for Hospital Episode Statistics data extraction

| Code | Code description |
| --- | --- |
| OPCS-4 codes for knee revision procedures | |
| O180 | Conversion from previous hybrid prosthetic replacement of knee joint using cement |
| O182 | Conversion to hybrid prosthetic replacement of knee joint using cement |
| O183 | Revision of hybrid prosthetic replacement of knee joint using cement |
| O184 | Attention to hybrid prosthetic replacement of knee joint using cement |
| W400 | Conversion from previous cemented total prosthetic replacement of knee joint |
| W402 | Conversion to total prosthetic replacement of knee joint using cement |
| W403 | Revision of total prosthetic replacement of knee joint using cement |
| W404 | Revision of one component of total prosthetic replacement of knee joint using cement |
| W410 | Conversion from previous uncemented total prosthetic replacement of knee joint |
| W412 | Conversion to total prosthetic replacement of knee joint not using cement |
| W413 | Revision of total prosthetic replacement of knee joint not using cement |
| W414 | Revision of one component of total prosthetic replacement of knee joint not using cement |
| W420 | Conversion from previous total prosthetic replacement of knee joint NEC |
| W422 | Conversion to total prosthetic replacement of knee joint NEC |
| W423 | Revision of total prosthetic replacement of knee joint NEC |
| W424* | Attention to total prosthetic replacement of knee joint NEC |
| W425 | Revision of one component of total prosthetic replacement of knee joint NEC |
| W522† | Conversion to prosthetic replacement of articulation of bone using cement NEC |
| W523† | Revision of prosthetic replacement of articulation of bone using cement NEC |
| W532† | Conversion to prosthetic replacement of articulation of bone not using cement NEC |
| W533† | Revision of prosthetic replacement of articulation of bone not using cement NEC |
| W542† | Conversion to prosthetic replacement of articulation of bone NEC |
| W543† | Revision of prosthetic replacement of articulation of bone NEC |
| W544*† | Attention to prosthetic replacement of articulation of bone NEC |
| W553† | Conversion to prosthetic interposition arthroplasty of joint |
| W564† | Conversion to interposition arthroplasty of joint NEC |
| W574† | Conversion to excision arthroplasty of joint |
| W582† | Revision of resurfacing arthroplasty of joint |
| W603† | Conversion to arthrodesis and extra-articular bone graft NEC |
| W613† | Conversion to arthrodesis and articular bone graft NEC |
| W641† | Conversion to arthrodesis and internal fixation NEC |
| W642† | Conversion to arthrodesis and external fixation NEC |
| OPCS-4 codes for laterality | |
| Z941 | Bilateral |
| Z942 | Left-sided |
| Z943 | Right-sided |
| ICD-10 codes for Infection | |
| T845 | Infection and inflammatory reaction due to internal joint prosthesis |
| T846 | Infection and inflammatory reaction due to internal fixation device [any site] |
| T847 | Infection and inflammatory reaction due to other internal orthopaedic prosthetic devices, implants and grafts |
| T814 | Infection following a procedure, not elsewhere classified |
| ICD-10 codes for fracture | |
| M966 | Fracture of bone following insertion of orthopaedic implant, joint prosthesis or bone plate |
| ICD-10 codes for mechanical complications | |
| T840 | Mechanical complication of internal joint prosthesis |
| T841 | Mechanical complication of internal fixation device of bones of limb |
| T842 | Mechanical complication of internal fixation device of other bones |
| T843 | Mechanical complication of other bone devices, implants and grafts |
| T844 | Mechanical complication of other internal orthopaedic devices, imnplants and grafts |
| ICD-10 codes for osteoarthritis/arthrosis | |
| M15- | Polyarthrosis |
| M17- | Gonarthrosis |
| M19- | Other arthrosis |

OPCS-4 = Office of Populations Censuses and Surveys Classification of Interventions and Procedures version 4. ICD-10 = International Statistical Classification of Diseases and Related Health Problems, tenth revision. * Where OPCS-4 codes Y032 (renewal of prosthesis in organ NOC) or Y037 (removal of prosthesis from organ NOC) were also used. † Where OPCS-4 codes O132 (knee NEC) or Z765 (lower end of femur NEC) or Z774 (upper end of tibia NEC) or Z787 (patella) or Z844 (patellofemoral joint) or Z845 (tibiofemoral joint) or Z846 (knee joint) or Z851 (upper tibiofibular joint) were used to identify knee as the body site.
