# Supplementary figures and images for: What is the impact of longer patient travel distances and times on perioperative outcomes following revision knee replacement: a retrospective observational study using data for England from Hospital Episode Statistics
Source: BMJ Open. 2025 May 6;15(5):e085201. doi: 10.1136/bmjopen-2024-085201 (PMC12056618; doi:10.1136/bmjopen-2024-085201)

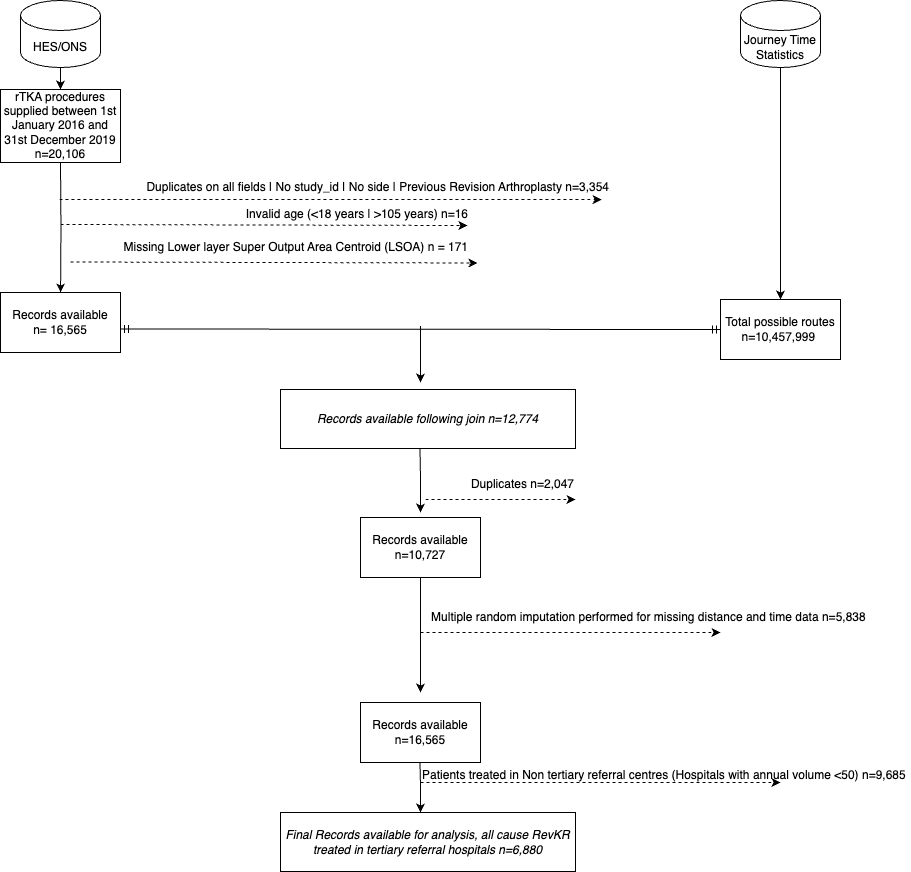

Supplement: online supplemental file 3 [file bmjopen-15-5-s003.jpg]

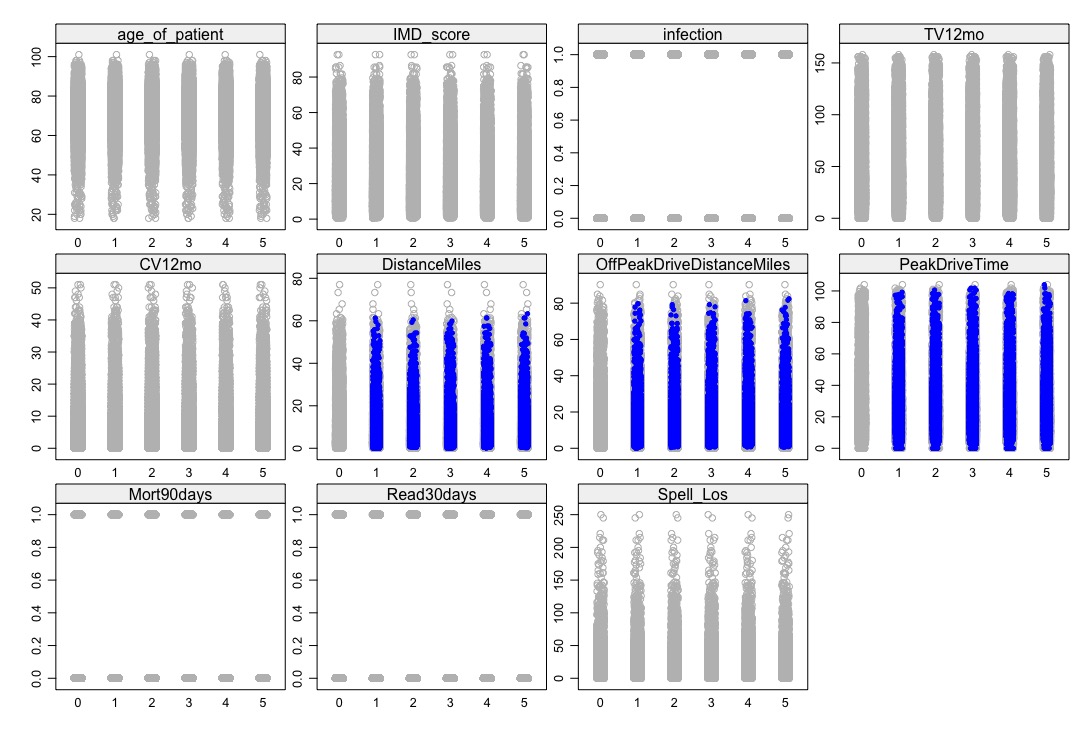

Supplement: online supplemental file 5 [file bmjopen-15-5-s005.jpeg]
