# Supplementary material for: What is the impact of longer patient travel distances and times on perioperative outcomes following revision knee replacement: a retrospective observational study using data for England from Hospital Episode Statistics
Source: BMJ Open. 2025 May 6;15(5):e085201. doi: 10.1136/bmjopen-2024-085201 (PMC12056618; doi:10.1136/bmjopen-2024-085201)
Supplement: online supplemental file 4 [file bmjopen-15-5-s004.pdf]

Supplementary material S4 – R Code

```
#Travel Times and Perioperative Outcomes in Revision Knee Replacement

setwd("/Users/alexandermatthews//OneDrive - University of Exeter/Alex Matthews
MD/Revision Knee Networks MD/Travel Times Analysis_/")


####Preparation of Data####
#load HES data

RTKA2023 <- read.csv("~/Desktop/RTKA 06-09-23 CSV.csv")

RTKA2023 <- read.csv("/Users/alexandermatthews//OneDrive - University of Exeter/Alex
Matthews MD/Revision Knee Networks MD/Travel Times Analysis_/RTKA 06-09-23
CSV.csv")



#table only shows first 50 columns but we know there are 51 columns. Write this generic
code to change preferences


rstudioapi::writeRStudioPreference("data_viewer_max_columns", 1000L)

#Some entried are blank but are read as real values and not missing data
#The table between age and sex shows three variables here
#The dataset contains non standard missing values that are not recognised as NA
#Replace empty strings with NA

RTKA2023[RTKA2023 == ""] <- NA

#Find number of incomplete cases in the data

missing_data <- colSums(is.na(RTKA2023))
print(missing_data)

#There are 14 entries with missing data only in the age group

#check how many incomplete entries in age of patient column

sum(!complete.cases(RTKA2023$age_of_patient))

#In case of missing values there are only 14 for age of patient
#Can use imputation based on mean age
#What is the mean age of the patients
```

```
mean(RTKA2023$age_of_patient, na.rm = TRUE)

#mean age excluding missing values is 70
summary(RTKA2023$age_of_patient, na.rm = TRUE)

#Check age is normally distributed

hist(RTKA2023$age_of_patient)

#Input mean for missing values for age

RTKA2023$age_of_patient[is.na(RTKA2023$age_of_patient)] <- 69.82


#Now check number of missing values

sum(!complete.cases(RTKA2023$age_of_patient))
#Now states 0 missing values

#There are other missing values for IMD decile
##In fact there are 439 IMD score missing values

sum(!complete.cases(RTKA2023$IMD_score))


hist(RTKA2023$IMD_score)
#IMD score is non normally distributed

summary(RTKA2023$IMD_score, na.rm = TURE)

#Median IMD score is 15.543

#Use imputation to impute median for missing value

RTKA2023$IMD_score[is.na(RTKA2023$IMD_score)] <- 15.543

#Check imputation complete

sum(!complete.cases(RTKA2023$IMD_score))

#Now showing 0 missing values

#Next attach IMD decile number 6 to the missing values. As a score of 15 equates to the 6th
decile

RTKA2023$IMD_decile[is.na(RTKA2023$IMD_decile)] <- 6
```

```
#Check duplicate entry spells

duplicates <- RTKA2023[duplicated(RTKA2023),]


#No duplicates in data


#Frequencies of revisions by volume

as.numeric(RTKA2023$TV12mo)


#frequencies of revisions by trust volume
table(RTKA2023$TVcat)

#Proportions by trust volume

prop.table(table(RTKA2023$TVcat))

#Some entried are blank but are read as real values and not missing data
#The table between age and sex shows three variables here
#The dataset contains non standard missing values that are not recognised as NA
#Replace empty strings with NA

RTKA2023[RTKA2023 == ""] <- NA

#Check this has registered

missing_data <- colSums(is.na(RTKA2023))
print(missing_data)


#Column with LSOA_2011_Code has 171 missing.

#LSOA is part of primary exposure variable, small number of missing cases. Decision to remove rows rather than estimate from imputation because factor variable and dependent on provider code. Multiple imputation was used later to estimate missing travel data for these multiple rows where LSOA and site code was availble

#Remove missing data in dataframe combined_data for column LSOA_2011_Code with missing fields = 171

RTKA2023<- RTKA2023[!is.na(RTKA2023$LSOA_2011_Code), ]
```

```
#16,565 patients before link with TRACC travel data

#Load Travel times data

TRAVELTIMES <- read.csv("~/Desktop/Drive time and Miles reference file.csv")

LSOAREF <- read.csv("~/Desktop/LSOA Matrix.csv")

LSOAREF <- read.csv("/Users/alexandermatthews//OneDrive - University of Exeter/Alex Matthews MD/Revision Knee Networks MD/Travel Times Analysis_/LSOA Matrix.csv")


#Join data but The data is too big so we need to do this using SQL

install.packages("RSQLite")
library(RSQLite)

con <- dbConnect(RSQLite::SQLite(),
            dbname = "mydatabase1.db")
dbWriteTable(con, "times", TRAVELTIMES)
dbWriteTable(con, "lsoa", LSOAREF)

query <- "
Select *
FROM times
JOIN lsoa ON times.LSOAName = lsoa.LSOA11NM"

result <- dbGetQuery(con, query)

#10million 457 thousand and 999 possible combinations

#Write Dataframes

write.csv(result, "~/Desktop/JOINLSOATRAVEL.csv")

result<- read.csv("/Users/alexandermatthews//OneDrive - University of Exeter/Alex Matthews MD/Revision Knee Networks MD/Travel Times Analysis_/JOINLSOATRAVEL.csv")


#####Now join this data to your revisions spreadsheet using key identifiers LSOA and Organisation site code

con <- dbConnect(RSQLite::SQLite(),
            dbname = "mydatabase1.db")
dbWriteTable(con, "revisions3", RTKA2023)
dbWriteTable(con, "travel3", result)
```

```
query <- "
Select *
FROM revisions3
JOIN travel3 ON revisions3.LSOA_2011_Code = travel3.LSOA11CD AND revisions3.Sitecode =
travel3.ProviderSiteCode"

result_join <- dbGetQuery(con, query)

#Number of patients following join 12,774


result1 <- result_join
#Check your data for missing values

missing_data <- colSums(is.na(result1))
print(missing_data)

#Check data for duplicates

duplicates <- RTKA2023[duplicated(RTKA2023$Epikey), ]


# Check for duplicates in the 'epikey' column
duplicates <- result1[duplicated(result1$Epikey), ]

#There are 2,047 duplicates

#Remove duplicates in result 1


# Remove duplicates: Keep only the first occurrence of each 'Epikey'
result1 <- result1[!duplicated(result1$Epikey), ]

#final dataframe is 10,727


write.csv(result1, "/Users/alexandermatthews//OneDrive - University of Exeter/Alex
Matthews MD/Revision Knee Networks MD/Travel Times Analysis_/FinalJOIN.csv")


####Prepare Outcomes, Exposure variable and co-variates ####

#Set up outcomes

#Replace NA's in the Read columns with N
```

```
result1$Read30 <- ifelse(is.na(result1$Read30), 'N', result1$Read30)
result1$Read90 <- ifelse(is.na(result1$Read90), 'N', result1$Read90)

result1$Read30days <- ifelse(result1$Read30 == "Y", 1, 0)
#readmission for 90 days
result1$Read90days <- ifelse(result1$Read90 == "Y", 1, 0)



#Set up your co-variates

result1$HFRS_Band = as.factor(result1$HFRS_Band)
result1$HFRS_Band = relevel(result1$HFRS_Band, ref = 'None')

result1$POD = as.factor(result1$POD)
result1$POD = relevel(result1$POD, ref = 'EL')

table(result1$POD)


#I've joined two dataframes based on a shared field. But some rows have not jointed

#Journey times statistics - 10,457,999 rows

#12,774 following join with revisions and travel data called "result1" but had duplicates
2,047 so remove these (duplicates due to slightly different latitude and longitude for same
Site codes in journey times statistics )

#Final results 1 following removal of duplicates is 10,727

#Original dataframe is 16,736 called RTKA2023 following removal of early revisions,
excluding missing LSOA was 16565

#Missing data for travel seen in 5,838 patients or 35% of patients

#Use multiple imputation to impute missing distance values for cases without join

#How many unmatched rows?

unmatched_rows <- RTKA2023[!(RTKA2023$Epikey %in% result1$Epikey), ]

#There are 5,838 unmatched rows

#I want to create a dataframe showing both matched and unmatched fields based on this.

# Identify columns that are in result1 but not in RTKA2023
missing_cols <- setdiff(names(result1), names(RTKA2023))
```

```
# Add missing columns to RTKA2023 with NA values
for (col in missing_cols) {
  RTKA2023[[col]] <- NA
}

# Ensure column order is the same as result1
RTKA2023 <- RTKA2023[, names(result1)]

# Identify unmatched rows
unmatched_rows <- RTKA2023[!(RTKA2023$Epikey %in% result1$Epikey), ]

# Combine matched rows (result1) with unmatched rows
combined_data <- rbind(result1, unmatched_rows)

duplicates <- combined_data[duplicated(combined_data$Epikey), ]

#0 duplicates

write.csv(combined_data, "/Users/alexandermatthews//OneDrive - University of
Exeter/Alex Matthews MD/Revision Knee Networks MD/Travel Times
Analysis_/FinalJOINCombined.csv")


combined_data <- read.csv("/Users/alexandermatthews//OneDrive - University of
Exeter/Alex Matthews MD/Revision Knee Networks MD/Travel Times
Analysis_/FinalJOINCombined.csv")

#Replace NA's in the Read columns with N

combined_data$Read30 <- ifelse(is.na(combined_data$Read30), 'N',
combined_data$Read30)


combined_data$Read30days <- ifelse(combined_data$Read30 == "Y", 1, 0)



#Now have dataframe displaying both matched and unmatched rows

missing_data <- colSums(is.na(combined_data))
print(missing_data)

#How many patients in high volume centres >49

combined_data$MRC <- ifelse(combined_data$TV12mo > 49, 1, 0)
```

```
nopatients <- subset(combined_data, MRC == 1)

#6880 patients

missing_data <- colSums(is.na(nopatients))
print(missing_data)

# Count unique levels of ProvCode
n_levels <- length(unique(nopatients$ProvCode))
cat("Number of unique providers (ProvCode):", n_levels, "\n")
#38 providers

#How many sites
# Count unique levels of ProvCode
n_levels <- length(unique(nopatients$Sitecode))
cat("Number of unique sites (Sitecode):", n_levels, "\n")

#187 sites

#rates of readmission 30 days

table(nopatients$Read30days)

#568/6880 8.3%

#rates of mortality at 90 days

table(nopatients$Mort90days)

#217/6880 3.2%

#Rates of length of stay above median. Remember median calculated across entire cohort

summary(combined_data$Spell_Los) #Median of 5

nopatients$Long_Los <- ifelse(nopatients$Spell_Los > 5, 1, 0)

table(nopatients$Long_Los)

#3421/6880 49.7%


#3157 travel data not available

#16,565 observations in entire dataframe not limited to teriatry referral centres

#CV12mo missing 71 cases. Imputation using median due to positive skew
```

hist(combined_data$CV12mo)

#mean age excluding missing values is 70
summary(combined_data$CV12mo, na.rm = TRUE)

#Input median of 6 for missing data

combined_data$CV12mo[is.na(combined_data$CV12mo)] <- 6

#Now need to use multiple imputation method to estimate travel data for columns "DistanceMiles", "OffPeakDriveDistanceMiles", "PeakDriveTimes' based on associated predictors:

#Refer to this resource "https://bookdown.org/mwheymans/bookmi/multiple-imputation.html#setting-the-imputation-methods"

#And this resource for context
https://dept.stat.lsa.umich.edu/~jerrick/courses/stat701/notes/mi.html

https://www.ebpi.uzh.ch/dam/jcr:dc0cef17-29c7-4e61-8d33-e690561ab7ae/mi_intro20191001.pdf (Advice on multi level modelling and imputation)

# Install packages if they are not already installed
install.packages(c("mice", "ggplot2", "naniar"))

# Load the packages
library(mice)
library(ggplot2)
library(naniar)

#assuming missing data is due to random chance, LSOA and SiteCode are related to the exposure but also include all other variables linked to your analysis
#Subset dataframe called combined_date with only with relevant columns: age_of_patient, sex, HFRS_Band IMD_Score, IMD_Decile, infection, TVcat, CVcat, SiteCode, ProvCode, FinY, DistanceMiles, OffPeakDriveDistanceMiles, PeakDriveTime, Mort90days, Read30, Spell_Los
#decision not to include site code and LSOA as likely not present in missing data "LSOA_2011_Code", "Sitecode"

# Specify the relevant columns I've included TV12mo as may be related to outcome, ProvCode for clustering,
relevant_columns <- c(
  "age_of_patient", "sex", "HFRS_Band", "IMD_score",
  "infection", "TV12mo", "CV12mo", "ProvCode", "FinY",

```
  "DistanceMiles", "OffPeakDriveDistanceMiles", "PeakDriveTime",
  "Mort90days", "Read30days", "Spell_Los"
)

# Subset the dataframe with only the relevant columns
subset_combined_data <- combined_data[, relevant_columns]

#Currently sex, HFRS_Band, TVCat, Sitecode, ProvCode, FinY are not incorporated in model
as character variables

#convert these to factors


# Convert variables to factors
subset_combined_data$sex <- as.factor(subset_combined_data$sex)
subset_combined_data$ProvCode <- as.factor(subset_combined_data$ProvCode)
subset_combined_data$FinY <- as.factor(subset_combined_data$FinY)
subset_combined_data$HFRS_Band <- as.factor(subset_combined_data$HFRS_Band)

subset_combined_data$Sitecode <- as.factor(subset_combined_data$Sitecode)
subset_combined_data$LSOA_2011_Code <-
as.factor(subset_combined_data$LSOA_2011_Code)



# Check the structure of the dataframe to confirm
str(subset_combined_data[, c("sex", "Sitecode", "ProvCode", "FinY", "HFRS_Band",
"LSOA_2011_Code")])


#visualise missing data

vis_miss(subset_combined_data)

#35% missing travel data

# Set the seed for reproducibility
set.seed(123)


# Perform Multiple Imputation

imp <- mice(subset_combined_data, m=5, method='pmm')

#Check for imputation values

imp$imp$OffPeakDriveDistanceMiles
```

#visualise imputed values

imp$imp

#Means of the imputed values

imp$chainMean

#What are the predictors

imp$predictorMatrix

#Plot imputation values against observed values.

my_plot <- stripplot(imp, col=c("grey", "blue"), pch = c(1, 20))

my_plot

#Guidelines for imputation model suggest all variables in the analysis should be included, inclusive of dependent or outcome variables

#Ensure TVCat is not a predictor variable

pred <-imp$predictorMatrix
pred["TVcat"] <- 0
pred

#Plot the convergence (how equal is the variance to the mean)

plot(imp)

#Stack the imputed values into a single dataset and include original data

imp2 <- complete(imp, "long", inc = TRUE)

#Save imp2

write.csv(imp2, "/Users/alexandermatthews//OneDrive - University of Exeter/Alex Matthews MD/Revision Knee Networks MD/Travel Times Analysis_/imp2.csv")

#Read it back in here:

imp2 <- read.csv("/Users/alexandermatthews//OneDrive - University of Exeter/Alex Matthews MD/Revision Knee Networks MD/Travel Times Analysis_/imp2.csv")

#Save as Supplemenatry figure

#Filter data by tertiary hospitals only

#But current guidelines suggest >49 is a high volume centre called a major revision centre and probably represents a unit with tertiary specialisation

```
imp2$MRC <- ifelse(imp2$TV12mo > 49, 1, 0)

tertiary_revisions <- subset(imp2, MRC == 1)

tertiary_revisions$Long_Los <- ifelse(tertiary_revisions$Spell_Los > 5, 1, 0)
```

#declare the imputed data to be mids again, the format MICE is expecting for regression analyses

```
tertiary_revisions <- as.mids(tertiary_revisions)
```

#Now run your regression model using a multivariable model

#A priori co-variates chosen based on evidence of predictors for readmission

####Primary Outcome 30 day readmission ####

#Exposure 1 - Distance Miles

```
library("lme4")

# Fit logistic regression on imputed datasets include ProvCode in fixed effects to account for clustering
m3.mi <- with(tertiary_revisions, glm(Read30days ~ DistanceMiles + IMD_score +
HFRS_Band +
              sex + age_of_patient + infection + TV12mo + CV12mo + FinY + ProvCode,
            family = "binomial"))


print(m3.mi)


# Pool results across imputed datasets
pooled_results <- pool(m3.mi)

# Summarize pooled results with confidence intervals
summary_pooled <- summary(pooled_results, conf.int = TRUE)
```

```
# Add Odds Ratios to the summary
summary_pooled$OR <- exp(summary_pooled$estimate)
summary_pooled$Lower_CI <- exp(summary_pooled$`2.5 %`)
summary_pooled$Upper_CI <- exp(summary_pooled$`97.5 %`)

# Display the final table with Odds Ratios and Confidence Intervals
print(summary_pooled)

#check for evidence of multicollinearity?

library(car)

# Use the long data including all imputations for VIF

tertiary_revisions <- complete(tertiary_revisions, "long", inc = TRUE)


# Fit a logistic regression model on the complete dataset
vif_model <- glm(Read30days ~ DistanceMiles + IMD_score + HFRS_Band +
                 sex + age_of_patient + infection + TV12mo + CV12mo + FinY + ProvCode,
                data = tertiary_revisions, family = "binomial")




# Calculate VIF
vif_values <- vif(vif_model)
print(vif_values)


#No evidence of multi-collinearity

#Is there a non linear relationship?

#Box Tidwell

#Recode back into correct format

tertiary_revisions <- as.mids(tertiary_revisions)

# Custom function to add log-transformed variable and interaction term
add_interaction <- function(data) {
  data$Log_DistanceMiles <- log(data$DistanceMiles)  # Add log-transformed variable
  data$Interaction <- data$DistanceMiles * data$Log_DistanceMiles  # Add interaction term
  return(data)
}
```

```
# Extract the long-format data including the original data
tertiary_revisions_modified <- complete(tertiary_revisions, action = "long", include = TRUE)

# Apply the transformation to each imputed dataset
tertiary_revisions_modified <- do.call("rbind",
                              lapply(split(tertiary_revisions_modified,
tertiary_revisions_modified$.imp),
                                    add_interaction))

# Convert back to mids object
tertiary_revisions_modified <- as.mids(tertiary_revisions_modified)

# Fit the logistic regression model with the interaction term
model <- with(tertiary_revisions_modified, glm(Read30days ~ DistanceMiles + Interaction,
data = tert
                                    family = binomial(link = "logit")))

# Pool the results
pooled_results <- pool(model)

# Summarize pooled results
summary_pooled <- summary(pooled_results, conf.int = TRUE)

# Extract the p-value for the interaction term
box_tidwell_p <- summary_pooled[summary_pooled$term == "Interaction", "p.value"]

# Print the p-value
print(box_tidwell_p)

# p value = 0.03 evidence of non linearity

#Are spline terms significant for DistanceMiles if using 3 knots, 4 knots and 5 knots

#Use data of all imputations in long format

tertiary_revisions <- complete(tertiary_revisions, "long", inc = TRUE)



# Load the required library
library(splines)

#AIC of non spline model

model <- glm(Read30days ~ DistanceMiles, data = tertiary_revisions, family = binomial)
summary(model)
```

```
#AIC 21862

# Define a function to fit and evaluate spline models with knots based on centiles
evaluate_centile_splines <- function(centiles, data) {
  # Calculate knots based on the specified centiles
  knots <- quantile(data$DistanceMiles, probs = centiles, na.rm = TRUE)

  # Fit a logistic regression model with natural splines using the calculated knots
  model_spline <- glm(Read30days ~ ns(DistanceMiles, knots = knots),
                      family = binomial(link = "logit"),
                      data = data)

  # Summarize the model
  summary_model <- summary(model_spline)

  # Extract p-values for the spline terms
  p_values <- summary_model$coefficients[-1, "Pr(>|z|)"]  # Exclude the intercept

  # Print the results
  cat("\nResults for centiles", centiles, ":\n")
  print(p_values)

  # Return the model and calculated knots for further inspection if needed
  return(list(model = model_spline, p_values = p_values, knots = knots))
}

# Example centile configurations for 3, 4, and 5 knots
centiles_3_knots <- c(0.05, 0.50, 0.95)  # 5th, 50th, and 95th percentiles
centiles_4_knots <- c(0.05, 0.35, 0.65, 0.95)  # Custom centiles for 4 knots
centiles_5_knots <- c(0.05, 0.25, 0.50, 0.75, 0.95)  # 5 knots centiles

# Evaluate models with centile-based knots using your dataset
results_3_knots <- evaluate_centile_splines(centiles = centiles_3_knots, data =
tertiary_revisions)
results_4_knots <- evaluate_centile_splines(centiles = centiles_4_knots, data =
tertiary_revisions)
results_5_knots <- evaluate_centile_splines(centiles = centiles_5_knots, data =
tertiary_revisions)

# Compare models with centile-based knots
cat("\nComparing models with different centile-based knots:\n")
anova(results_3_knots$model, results_4_knots$model, results_5_knots$model, test =
"Chisq")

# Print the calculated knot locations for each model
cat("\nKnot locations for 3 knots:\n")
```

```
print(results_3_knots$knots)
cat("\nKnot locations for 4 knots:\n")
print(results_4_knots$knots)
cat("\nKnot locations for 5 knots:\n")
print(results_5_knots$knots)

#AIC better fit 21806
#Model with 3 knots, significant terms but greater knots do not improve the model fit. Non linear relationship is evident and should be modelled with splines





#Prepare predictors for model prediction


#you need to ensure that the predicted probabilities align with the corresponding observations
#Explore the data for missing values
sum(!complete.cases(tertiary_revisions$DistanceMiles))
#Unimputed dataset is missing, so exclude these

tertiary_revisions <- tertiary_revisions[!is.na(tertiary_revisions$DistanceMiles),]


sum(!complete.cases(tertiary_revisions$sex))

sum(!complete.cases(tertiary_revisions$Read30days))

sum(!complete.cases(tertiary_revisions$HFRS_Band))

sum(!complete.cases(tertiary_revisions$IMD_score))

sum(!complete.cases(tertiary_revisions$infection))

#Currently infection as numeric - ensure is factor

tertiary_revisions$infection <- as.factor(tertiary_revisions$infection)
tertiary_revisions$HFRS_Band <- as.factor(tertiary_revisions$HFRS_Band)
tertiary_revisions$sex <- as.factor(tertiary_revisions$sex)
tertiary_revisions$FinY <- as.factor(tertiary_revisions$FinY)
tertiary_revisions$ProvCode <- as.factor(tertiary_revisions$ProvCode)
tertiary_revisions$DistanceMiles <- as.numeric(tertiary_revisions$DistanceMiles)
tertiary_revisions$age_of_patient <- as.numeric(tertiary_revisions$age_of_patient)
tertiary_revisions$IMD_score <- as.numeric(tertiary_revisions$IMD_score)
tertiary_revisions$TV12mo <- as.numeric(tertiary_revisions$TV12mo)
```

```
tertiary_revisions$CV12mo <- as.numeric(tertiary_revisions$CV12mo)

#Run spline model with adjusted data excluding missing data
library(splines)
# For example, let's say you want 3 knots at specific percentiles
knots <- quantile(tertiary_revisions$DistanceMiles, probs = c(0.05, 0.50, 0.95), na.rm =
TRUE)
print(knots)
#Knots at 53, 69 and 84
spline_terms <- ns(tertiary_revisions$DistanceMiles, knots = knots)



model_with_custom_splines <- glm(Read30days ~ ns(DistanceMiles, knots = knots) +
HFRS_Band + IMD_score +
                            sex + age_of_patient + infection + TV12mo + CV12mo + FinY + ProvCode,
                          family = "binomial", data = tertiary_revisions)


summary(model_with_custom_splines)

#Generate a sequence of mean unit values for predicting

DistanceMiles_range <- seq(min(tertiary_revisions$DistanceMiles),
max(tertiary_revisions$DistanceMiles), length.out = 100)

new_data <- expand.grid(
  DistanceMiles = DistanceMiles_range,
  sex = levels(tertiary_revisions$sex),  # Ensure it takes all factor levels
  age_of_patient = mean(tertiary_revisions$age_of_patient, na.rm = TRUE),
  HFRS_Band = levels(tertiary_revisions$HFRS_Band),  # Ensuring correct factor levels
  IMD_score = mean(tertiary_revisions$IMD_score, na.rm = TRUE),
  FinY = levels(tertiary_revisions$FinY),  # Ensuring correct factor levels
  CV12mo = mean(tertiary_revisions$CV12mo, na.rm = TRUE),
  TV12mo = mean(tertiary_revisions$TV12mo, na.rm = TRUE),
  ProvCode = levels(tertiary_revisions$ProvCode),  # Ensuring correct factor levels
  infection = levels(tertiary_revisions$infection)  # Ensuring correct factor levels
)

# Create a new dataset with a range of distances and miles and all other predictor variables
new_data <- expand.grid(DistanceMiles = DistanceMiles_range,
                        sex = unique(tertiary_revisions$sex),
                        age_of_patient = mean(tertiary_revisions$age_of_patient),
                        HFRS_Band = unique(tertiary_revisions$HFRS_Band),
                        IMD_score = mean(tertiary_revisions$IMD_score),
                        FinY = unique(tertiary_revisions$FinY),
                        CV12mo = mean(tertiary_revisions$CV12mo),
```

```
                    TV12mo = mean(tertiary_revisions$TV12mo),
                    infection = unique(tertiary_revisions$infection))


# Align the levels of ProvCode in new_data to match the training data
new_data$ProvCode <- factor(new_data$ProvCode, levels =
levels(tertiary_revisions$ProvCode))

# Align the levels of all relevant categorical variables
new_data$HFRS_Band <- factor(new_data$HFRS_Band, levels =
levels(tertiary_revisions$HFRS_Band))
new_data$sex <- factor(new_data$sex, levels = levels(tertiary_revisions$sex))
new_data$FinY <- factor(new_data$FinY, levels = levels(tertiary_revisions$FinY))
new_data$infection <- factor(new_data$infection, levels =
levels(tertiary_revisions$infection))

#Factors are consistent with model

levels(new_data$HFRS_Band)
levels(tertiary_revisions$HFRS_Band)

levels(new_data$sex)
levels(tertiary_revisions$sex)

levels(new_data$FinY)
levels(tertiary_revisions$FinY)

levels(new_data$ProvCode)
levels(tertiary_revisions$ProvCode)

levels(new_data$infection)
levels(tertiary_revisions$infection)

# Check levels of ProvCode in both datasets
setdiff(levels(new_data$ProvCode), levels(tertiary_revisions$ProvCode))  # Levels in
new_data but not in tertiary_revisions
setdiff(levels(tertiary_revisions$ProvCode), levels(new_data$ProvCode))  # Levels in
tertiary_revisions but not in new_data

new_data$ProvCode <- droplevels(new_data$ProvCode)
# Check for missing values in factor variables
sum(is.na(new_data$ProvCode))  # Number of missing values in ProvCode

# Ensure that ProvCode is a factor
new_data$ProvCode <- factor(new_data$ProvCode, levels =
levels(tertiary_revisions$ProvCode))
```

```r
# Now try the prediction again
predicted_probs <- predict(model_with_custom_splines, newdata = new_data, type = "response")




# Combine mean_unit_range and predicted_probs into a data frame
plot_data <- data.frame(DistanceMiles = DistanceMiles_range, predicted_prob = predicted_probs)

#Calculate 95% confidence intervals

# Obtain predicted values and standard errors for the new data
predictions <- predict(model_with_custom_splines, newdata = new_data, type = "link", se.fit = TRUE)

# Calculate the confidence intervals for the log-odds scale (link scale)
# Use a 95% confidence level (z-value = 1.96 for a 95% CI)
z_value <- 1.96
log_odds_lower <- predictions$fit - z_value * predictions$se.fit
log_odds_upper <- predictions$fit + z_value * predictions$se.fit

# Convert the log-odds confidence intervals to probabilities
# First, apply the inverse link function (logistic function) to the log-odds
lower_prob <- plogis(log_odds_lower)
upper_prob <- plogis(log_odds_upper)

# Combine the predicted probabilities and their confidence intervals into a data frame
plot_data <- data.frame(
  DistanceMiles = new_data$DistanceMiles,
  predicted_prob = plogis(predictions$fit),  # Logistic transformation of the link
  ci_lower = lower_prob,
  ci_upper = upper_prob
)



# Combine mean_unit_range, predicted_probs, ci_lower, and ci_upper into plot_data
plot_data <- data.frame(DistanceMiles = DistanceMiles_range,
                        predicted_prob = predicted_probs,
                        ci_lower = boot_results$ci_lower,
                        ci_upper = boot_results$ci_upper)

library(ggplot2)
# Plot the spline curve with confidence intervals
ggplot(plot_data, aes(x = DistanceMiles)) +
```

```
  geom_line(aes(y = predicted_prob), color = "blue", size = 1) +
  geom_ribbon(aes(ymin = ci_lower, ymax = ci_upper), fill = "blue", alpha = 0.2) +
  labs(x = "Distance (Miles)", y = "Predicted Probability Readmission at 30 days") +
  theme_minimal()

library(dplyr)

# Group by mean_unit and calculate mean predicted_prob and corresponding confidence
intervals
mean_data <- plot_data %>%
  group_by(DistanceMiles) %>%
  summarise(
    mean_predicted_prob = mean(predicted_prob, na.rm = TRUE),
    mean_ci_lower = mean(ci_lower, na.rm = TRUE),
    mean_ci_upper = mean(ci_upper, na.rm = TRUE)
  )


# Define specific breaks (e.g., 25, 50, 75, ..., up to the maximum)
breaks_seq <- seq(0, max(mean_data$DistanceMiles, na.rm = TRUE), by = 5)

library(ggplot2)
# Plot with specified increments on x-axis
ggplot(mean_data, aes(x = DistanceMiles, y = mean_predicted_prob)) +
  geom_point() +  # Add points for mean_predicted_prob
  geom_line() +   # Connect points with a line
  geom_ribbon(aes(ymin = mean_ci_lower, ymax = mean_ci_upper), fill = "blue", alpha =
0.2) +  # Add ribbon for confidence intervals
  labs(x = "Travel Distance (Miles)", y = "Mean Predicted Probability for readmission at 30
days", title = "Spline curve predicted probability of readmission at 30 days by patient travel
distance") +
  scale_x_continuous(limits = c(0, max(mean_data$DistanceMiles, na.rm = TRUE)), breaks =
breaks_seq) +
  theme_minimal() +
  theme(
    axis.title.x = element_text(size = 14),  # Increase x-axis title font size
    axis.title.y = element_text(size = 14),  # Increase y-axis title font size
    axis.text.x = element_text(size = 12),   # Increase x-axis tick label font size
    axis.text.y = element_text(size = 12),   # Increase y-axis tick label font size
    plot.title = element_text(size = 16, hjust = 0.5)  # Increase plot title font size and center it
  )


#Spline curve does appear to show the predicted probability of emergency readmission at
30 days increases with travel distance but wide confidence intervals

#Model Distance Miles and 30 day readmission with 3 knot splines
```

```
####First Imputation and descriptive stats####

#Use first imputed data for clinical and demographic characteristic summary

#complete_data is the first imputation

# Count unique levels of ProvCode
n_levels <- length(unique(complete_data$ProvCode))
cat("Number of unique providers (ProvCode):", n_levels, "\n")


# Count unique levels of sites
n_levels <- length(unique(complete_data))
cat("Number of unique providers (ProvCode):", n_levels, "\n")

# Count unique levels of ProvCode
n_levels <- length(unique(tertiary_revisions$ProvCode))
cat("Number of unique providers (ProvCode):", n_levels, "\n")

#38 unique providers



#Number of sites

# Count unique levels of Sites but need to use original dataframe as sites not included in
imputation analysis

#Find all those attending tertirary referral centre from original data
tertiary_all <- subset(combined_data, MRC == 1)

#Find number of sites
n_levels <- length(unique(tertiary_all$Sitecode))
cat("Number of unique providers (Sites):", n_levels, "\n")

#187 sites

#Back to first imputation dataset. Calculate median number of miles straight line distance

summary(complete_data$DistanceMiles)

#Median is 7.1 IQR is 3.9 to 12.7. Range 0 to 77.1 miles.
```

```
#Driving distances

summary(complete_data$OffPeakDriveDistanceMiles)

#Median 10.4 miles, IQR is 5.8 to 18.3 miles

#Calculate median driving times

summary(complete_data$PeakDriveTime)

#Median is 27 minutes IQR is 18.4 to 38.4. Maximum 104 minutes


#Create travel time quintile variable

quintiles <- quantile(complete_data$DistanceMiles, probs = seq(0,1,0.2), na.rm=TRUE)

complete_data$distancequintile <- cut(complete_data$DistanceMiles, breaks = quintiles,
labels = c("Q1", "Q2", "Q3", "Q4", "Q5"), include.lowest = TRUE)

#Tabulate descriptive stats

hist(tertiary_all$Spell_Los)
summary(tertiary_all$Spell_Los)

# Total number of revisions
total_revisions <- nrow(complete_data)

# Create a summary table
summary_stats <- complete_data %>%
  group_by(distancequintile) %>%
  summarise(
    # Count of observations
    Count = n(),

    # Distinct Providers
    Distinct_Units = n_distinct(ProvCode),
    Total_Distinct_Units = n_distinct(complete_data$ProvCode),
    Distinct_Units_Percent = (Distinct_Units / Total_Distinct_Units) * 100,

    #Median distance

    Distance_LowerQuartile = quantile(DistanceMiles, 0.25, na.rm = TRUE),
    Distance_Median = median(DistanceMiles, na.rm = TRUE),
    Distance_UpperQuartile = quantile(DistanceMiles, 0.75, na.rm = TRUE),
```

```
#Mean driving time
DrivingTime_LowerQuartile = quantile(PeakDriveTime, 0.25, na.rm = TRUE),
DrivingTime_Median = median(PeakDriveTime, na.rm = TRUE),
DdrivingTime_UpperQuartile = quantile(PeakDriveTime, 0.75, na.rm = TRUE),



# Age: Mean and standard deviation
Age_Mean = mean(age_of_patient, na.rm = TRUE),
Age_SD = sd(age_of_patient, na.rm = TRUE),

# Age: Mean ± SD (concatenated)
Age_Mean_SD = paste(round(mean(age_of_patient, na.rm = TRUE), 2), "±",
round(sd(age_of_patient, na.rm = TRUE), 2)),


# Gender: frequency and percentage
Female_Freq = sum(sex == "Female", na.rm = TRUE),
Female_Percent = sum(sex == "Female", na.rm = TRUE) / n() * 100,
Male_Freq = sum(sex == "Male", na.rm = TRUE),
Male_Percent = sum(sex == "Male", na.rm = TRUE) / n() * 100,

# ASA: frequency and percentage for each level
HFRS_None_Freq = sum(HFRS_Band == "None", na.rm = TRUE),
HFRS_None_Percent = sum(HFRS_Band == "None", na.rm = TRUE) / n() * 100,
HFRS_Mild_Freq = sum(HFRS_Band == "Mild", na.rm = TRUE),
HFRS_Mild_Percent = sum(HFRS_Band == "Mild", na.rm = TRUE) / n() * 100,
HFRS_Moderate_Freq = sum(HFRS_Band == "Moderate", na.rm = TRUE),
HFRS_Moderate_Percent = sum(HFRS_Band == "Moderate", na.rm = TRUE) / n() * 100,
HFRS_Severe_Freq = sum(HFRS_Band == "Severe", na.rm = TRUE),
HFRS_Severe_Percent = sum(HFRS_Band == "Severe", na.rm = TRUE) / n() * 100,


#Infection

Infection_Freq = sum(infection == "1", na.rm = TRUE),
Infection_Percent = sum(infection == "1", na.rm = TRUE) / n() * 100,


# Year: frequency and percentage for each year from 2009 to 2019
Year_2015_2016_Freq = sum(FinY == "2015/16", na.rm = TRUE),
Year_2015_2016_Percent = sum(FinY == "2015/16", na.rm = TRUE) / n() * 100,
Year_2016_2017_Freq = sum(FinY == "2016/17", na.rm = TRUE),
Year_2016_2017_Percent = sum(FinY == "2016/17", na.rm = TRUE) / n() * 100,
Year_2017_2018_Freq = sum(FinY == "2017/18", na.rm = TRUE),
Year_2017_2018_Percent = sum(FinY == "2017/18", na.rm = TRUE) / n() * 100,
Year_2018_2019_Freq = sum(FinY == "2018/19", na.rm = TRUE),
```

```
    Year_2018_2019_Percent = sum(FinY == "2018/19", na.rm = TRUE) / n() * 100,
    Year_2019_2020_Freq = sum(FinY== "2019/20", na.rm = TRUE),
    Year_2019_2020_Percent = sum(FinY == "2019/20", na.rm = TRUE) / n() * 100,


    # Median Surgeon Volume: lower quartile, median, and upper quartile
    Surgeon_LowerQuartile = quantile(CV12mo, 0.25, na.rm = TRUE),
    Surgeon_Median = median(CV12mo, na.rm = TRUE),
    Surgeon_UpperQuartile = quantile(CV12mo, 0.75, na.rm = TRUE),


    #Median hospital volume

    Hospital_LowerQuartile = quantile(TV12mo, 0.25, na.rm = TRUE),
    Hospital_Median = median(TV12mo, na.rm = TRUE),
    Hospital_UpperQuartile = quantile(TV12mo, 0.75, na.rm = TRUE),


    #Median IMD Score

    IMD_LowerQuartile = quantile(IMD_score, 0.25, na.rm = TRUE),
    IMD_Median = median(IMD_score, na.rm = TRUE),
    IMD_UpperQuartile = quantile(IMD_score, 0.75, na.rm = TRUE),

  )



# Print the summary table
print(summary_stats)

write.csv(summary_stats, "/Users/alexandermatthews//OneDrive - University of
Exeter/Alex Matthews MD/Revision Knee Networks MD/Travel Times
Analysis_/Summary_stats.csv")




####Cluster Variable ####

# Compute the mean outcome for each cluster
library(dplyr)
prov_means <- tertiary_revisions %>%
  group_by(ProvCode) %>%
  summarize(mean_outcome = mean(Read30days, na.rm = TRUE))

# Plot variability
```

```
boxplot(mean_outcome ~ ProvCode, data = prov_means, xlab = "ProvCode", ylab = "Mean
Outcome")

# Summary statistics of variability
summary(prov_means$mean_outcome)

#There is evidence of variability between providers


# Fit logistic regression on imputed datasets
m3.mi <- with(tertiary_revisions, glmer(Read30days ~ DistanceMiles + IMD_score +
HFRS_Band +
                          sex + age_of_patient + infection + TV12mo + CV12mo + FinY + (1 |
ProvCode),
                        family = "binomial"))


print(m3.mi)

#Including ProvCode as a random effect was tested but led to convergence issues likely due
to numerical instability between providers so a decision was made to accept the fixed
effects model which may account for clustering at the provider level but is a limitation of
the study


#Was travel distance strongly correlated with IMD_score or age?



#Next do a Spearman's rank correlation between travel distance and age, and then for
travel distance and IMD score

imp2$MRC <- ifelse(imp2$TV12mo > 49, 1, 0)

tertiary_revisions <- subset(imp2, MRC == 1)


write.csv(tertiary_revisions, "/Users/alexandermatthews//OneDrive - University of
Exeter/Alex Matthews MD/Revision Knee Networks MD/Travel Times
Analysis_/tertiary_revisions.csv")


tertiary_revisions <- as.mids(tertiary_revisions)

tertiary_revisions$age_of_patient <-
as.numeric(as.character(tertiary_revisions$age_of_patient))
```

```
tertiary_revisions$DistanceMiles <-
as.numeric(as.character(tertiary_revisions$DistanceMiles))



#Age and travel distance, Cannot pool the results based on the multiple imputations as cor
test not compatible.Therefore stack all imputations together and calculate correlation


# Scatterplot with linear regression line
plot(tertiary_revisions$age_of_patient, tertiary_revisions$DistanceMiles,
     main = "Scatterplot of Age of Patient vs DistanceMiles",
     xlab = "Age of Patient", ylab = "DistanceMiles",
     pch = 19, col = "blue")

# Add a linear trendline
abline(lm(DistanceMiles ~ age_of_patient, data = tertiary_revisions), col = "red", lwd = 2)

# Calculate Spearman's rank correlation
spearman_test <- cor.test(tertiary_revisions$age_of_patient,
tertiary_revisions$DistanceMiles, method = "spearman")

# Extract rho and p-value
rho <- round(spearman_test$estimate, 2)
p_value <- spearman_test$p.value
p_value_text <- ifelse(p_value < 0.05, "<0.05", paste0("=", round(p_value, 3)))

# Add a legend with Spearman's rank correlation information
legend("topright", legend = paste("Spearman's Rank Correlation:\n",
                                  "rho =", rho, ", p-value", p_value_text),
       col = c("blue", "red"), lty = c(NA, 1), pch = c(19, NA), lwd = c(NA, 2), bty = "n")


#IMD score and travel distance

# Scatterplot with trendline
plot(tertiary_revisions$IMD_score, tertiary_revisions$DistanceMiles,
     main = "Scatterplot of IMD_score vs DistanceMiles",
     xlab = "IMD_score", ylab = "DistanceMiles",
     pch = 19, col = "blue")

# Add a linear trendline (for visualizing the general trend)
abline(lm(DistanceMiles ~ IMD_score, data = tertiary_revisions), col = "red", lwd = 2)

# Calculate Spearman's rank correlation
spearman_test <- cor.test(tertiary_revisions$IMD_score, tertiary_revisions$DistanceMiles,
method = "spearman")
```

```
# Extract rho and p-value
rho <- round(spearman_test$estimate, 2)
p_value <- spearman_test$p.value
p_value_text <- ifelse(p_value < 0.05, "<0.05", paste0("=", round(p_value, 3)))

# Add a legend with Spearman's rank correlation information
legend("topright", legend = paste("Spearman's Rank Correlation:\n",
                          "rho =", rho, ", p-value", p_value_text),
      col = c("blue", "red"), lty = c(NA, 1), pch = c(19, NA), lwd = c(NA, 2), bty = "n")

#Exposure 2 - OffPeakDriveDistanceMiles

library("lme4")

# Fit logistic regression on imputed datasets include ProvCode in fixed effects to account for
clustering
m3.mi <- with(tertiary_revisions, glm(Read30days ~ OffPeakDriveDistanceMiles +
IMD_score + HFRS_Band +
                              sex + age_of_patient + infection + TV12mo + CV12mo + FinY +
ProvCode,
                             family = "binomial"))


print(m3.mi)


# Pool results across imputed datasets
pooled_results <- pool(m3.mi)

# Summarize pooled results with confidence intervals
summary_pooled <- summary(pooled_results, conf.int = TRUE)

# Add Odds Ratios to the summary
summary_pooled$OR <- exp(summary_pooled$estimate)
summary_pooled$Lower_CI <- exp(summary_pooled$`2.5 %`)
summary_pooled$Upper_CI <- exp(summary_pooled$`97.5 %`)

# Display the final table with Odds Ratios and Confidence Intervals
print(summary_pooled)

#check for evidence of multicollinearity?

library(car)

# Use the first imputed dataset for the VIF calculation
complete_data <- complete(tertiary_revisions, 1)
```

```
# Fit a logistic regression model on the complete dataset
vif_model <- glm(Read30days ~ OffPeakDriveDistanceMiles + IMD_score + HFRS_Band +
                 sex + age_of_patient + infection + TV12mo + CV12mo + FinY + ProvCode,
               data = complete_data, family = "binomial")

# Calculate VIF
vif_values <- vif(vif_model)
print(vif_values)


#No evidence of multi-collinearity

#Is there a non linear relationship?


# Custom function to add log-transformed variable and interaction term
add_interaction <- function(data) {
  data$Log_OffPeakDriveDistanceMiles <- log(data$OffPeakDriveDistanceMiles)  # Add log-
transformed variable
  data$Interaction <- data$OffPeakDriveDistanceMiles *
data$Log_OffPeakDriveDistanceMiles  # Add interaction term
  return(data)
}

# Extract the long-format data including the original data
tertiary_revisions_modified <- complete(tertiary_revisions, action = "long", include = TRUE)

# Apply the transformation to each imputed dataset
tertiary_revisions_modified <- do.call("rbind",
                                lapply(split(tertiary_revisions_modified,
tertiary_revisions_modified$.imp),
                                    add_interaction))

# Convert back to mids object
tertiary_revisions_modified <- as.mids(tertiary_revisions_modified)

# Fit the logistic regression model with the interaction term
model <- with(tertiary_revisions_modified, glm(Read30days ~ OffPeakDriveDistanceMiles +
Interaction,
                                    family = binomial(link = "logit")))

# Pool the results
pooled_results <- pool(model)

# Summarize pooled results
summary_pooled <- summary(pooled_results, conf.int = TRUE)
```

```
# Extract the p-value for the interaction term
box_tidwell_p <- summary_pooled[summary_pooled$term == "Interaction", "p.value"]

# Print the p-value
print(box_tidwell_p)

# p value = 0.05. There is no evidence of non linearity

#Exposure 3 - PeakDriveTime



library("lme4")

# Fit logistic regression on imputed datasets include ProvCode in fixed effects to account for
clustering
m3.mi <- with(tertiary_revisions, glm(Read30days ~ PeakDriveTime + IMD_score +
HFRS_Band +
                          sex + age_of_patient + infection + TV12mo + CV12mo + FinY +
ProvCode,
                         family = "binomial"))


print(m3.mi)


# Pool results across imputed datasets
pooled_results <- pool(m3.mi)

# Summarize pooled results with confidence intervals
summary_pooled <- summary(pooled_results, conf.int = TRUE)

# Add Odds Ratios to the summary
summary_pooled$OR <- exp(summary_pooled$estimate)
summary_pooled$Lower_CI <- exp(summary_pooled$`2.5 %`)
summary_pooled$Upper_CI <- exp(summary_pooled$`97.5 %`)

# Display the final table with Odds Ratios and Confidence Intervals
print(summary_pooled)

#check for evidence of multicollinearity?

library(car)

# Use the first imputed dataset for the VIF calculation
complete_data <- complete(tertiary_revisions, 1)
```

```
# Fit a logistic regression model on the complete dataset
vif_model <- glm(Read30days ~ PeakDriveTime + IMD_score + HFRS_Band +
                  sex + age_of_patient + infection + TV12mo + CV12mo + FinY + ProvCode,
                data = complete_data, family = "binomial")

# Calculate VIF
vif_values <- vif(vif_model)
print(vif_values)


#No evidence of multi-collinearity

#Is there a non linear relationship?



# Custom function to add log-transformed variable and interaction term
add_interaction <- function(data) {
  data$Log_PeakDriveTime <- log(data$PeakDriveTime)  # Add log-transformed variable
  data$Interaction <- data$PeakDriveTime * data$Log_PeakDriveTime  # Add interaction
term
  return(data)
}

# Extract the long-format data including the original data
tertiary_revisions_modified <- complete(tertiary_revisions, action = "long", include = TRUE)

# Apply the transformation to each imputed dataset
tertiary_revisions_modified <- do.call("rbind",
                                   lapply(split(tertiary_revisions_modified,
tertiary_revisions_modified$.imp),
                                         add_interaction))

# Convert back to mids object
tertiary_revisions_modified <- as.mids(tertiary_revisions_modified)

# Fit the logistic regression model with the interaction term
model <- with(tertiary_revisions_modified, glm(Read30days ~ PeakDriveTime + Interaction,
                                          family = binomial(link = "logit")))

# Pool the results
pooled_results <- pool(model)

# Summarize pooled results
summary_pooled <- summary(pooled_results, conf.int = TRUE)
```

```
# Extract the p-value for the interaction term
box_tidwell_p <- summary_pooled[summary_pooled$term == "Interaction", "p.value"]

# Print the p-value
print(box_tidwell_p)

# p value = 0.13 not evidence of non linearity





####Secondary Outcome mortality 90 days ####


#Exposure 1 - Distance Miles

library("lme4")

# Fit logistic regression on imputed datasets include ProvCode in fixed effects to account for clustering
m3.mi <- with(tertiary_revisions, glm(Mort90days ~ DistanceMiles + IMD_score +
HFRS_Band +
                                      sex + age_of_patient + infection + TV12mo + CV12mo + FinY +
ProvCode,
                                    family = "binomial"))


print(m3.mi)


# Pool results across imputed datasets
pooled_results <- pool(m3.mi)

# Summarize pooled results with confidence intervals
summary_pooled <- summary(pooled_results, conf.int = TRUE)

# Add Odds Ratios to the summary
summary_pooled$OR <- exp(summary_pooled$estimate)
summary_pooled$Lower_CI <- exp(summary_pooled$`2.5 %`)
summary_pooled$Upper_CI <- exp(summary_pooled$`97.5 %`)

# Display the final table with Odds Ratios and Confidence Intervals
print(summary_pooled)

#check for evidence of multicollinearity?
```

```
library(car)

# Use the first imputed dataset for the VIF calculation
complete_data <- complete(tertiary_revisions, 1)

# Fit a logistic regression model on the complete dataset
vif_model <- glm(Mort90days ~ DistanceMiles + IMD_score + HFRS_Band +
                  sex + age_of_patient + infection + TV12mo + CV12mo + FinY + ProvCode,
                data = complete_data, family = "binomial")

# Calculate VIF
vif_values <- vif(vif_model)
print(vif_values)


#No evidence of multi-collinearity


#Is there evidence of non linearity?

library(mice)

tertiary_revisions <- as.mids(tertiary_revisions)


# Custom function to add log-transformed variable and interaction term
add_interaction <- function(data) {
  data$Log_DistanceMiles <- log(data$DistanceMiles)  # Add log-transformed variable
  data$Interaction <- data$DistanceMiles * data$Log_DistanceMiles  # Add interaction term
  return(data)
}

# Extract the long-format data including the original data
tertiary_revisions_modified <- complete(tertiary_revisions, action = "long", include = TRUE)

# Apply the transformation to each imputed dataset
tertiary_revisions_modified <- do.call("rbind",
                                lapply(split(tertiary_revisions_modified,
tertiary_revisions_modified$.imp),
                                       add_interaction))

# Convert back to mids object
tertiary_revisions_modified <- as.mids(tertiary_revisions_modified)

# Fit the logistic regression model with the interaction term
model <- with(tertiary_revisions_modified, glm(Mort90days ~ DistanceMiles + Interaction,
                                       family = binomial(link = "logit")))
```

```
# Pool the results
pooled_results <- pool(model)

# Summarize pooled results
summary_pooled <- summary(pooled_results, conf.int = TRUE)

# Extract the p-value for the interaction term
box_tidwell_p <- summary_pooled[summary_pooled$term == "Interaction", "p.value"]

# Print the p-value
print(box_tidwell_p)


# P value 0.95

#Exposure 2 - OffPeakDriveDistanceMiles



library("lme4")

# Fit logistic regression on imputed datasets include ProvCode in fixed effects to account for
clustering
m3.mi <- with(tertiary_revisions, glm(Mort90days ~ OffPeakDriveDistanceMiles +
IMD_score + HFRS_Band +
                          sex + age_of_patient + infection + TV12mo + CV12mo + FinY +
ProvCode,
                         family = "binomial"))


print(m3.mi)


# Pool results across imputed datasets
pooled_results <- pool(m3.mi)

# Summarize pooled results with confidence intervals
summary_pooled <- summary(pooled_results, conf.int = TRUE)

# Add Odds Ratios to the summary
summary_pooled$OR <- exp(summary_pooled$estimate)
summary_pooled$Lower_CI <- exp(summary_pooled$`2.5 %`)
summary_pooled$Upper_CI <- exp(summary_pooled$`97.5 %`)

# Display the final table with Odds Ratios and Confidence Intervals
print(summary_pooled)
```

```
#check for evidence of multicollinearity?

library(car)

# Use the first imputed dataset for the VIF calculation
complete_data <- complete(tertiary_revisions, 1)

# Fit a logistic regression model on the complete dataset
vif_model <- glm(Read30days ~ OffPeakDriveDistanceMiles + IMD_score + HFRS_Band +
                 sex + age_of_patient + infection + TV12mo + CV12mo + FinY + ProvCode,
                data = complete_data, family = "binomial")

# Calculate VIF
vif_values <- vif(vif_model)
print(vif_values)


#No evidence of multi-collinearity

#Is there evidence of non linearity?

tertiary_revisions <- as.mids(tertiary_revisions)

# Custom function to add log-transformed variable and interaction term
add_interaction <- function(data) {
  data$Log_OffPeakDriveDistanceMiles <- log(data$OffPeakDriveDistanceMiles)  # Add log-
transformed variable
  data$Interaction <- data$OffPeakDriveDistanceMiles *
data$Log_OffPeakDriveDistanceMiles  # Add interaction term
  return(data)
}

# Extract the long-format data including the original data
tertiary_revisions_modified <- complete(tertiary_revisions, action = "long", include = TRUE)

# Apply the transformation to each imputed dataset
tertiary_revisions_modified <- do.call("rbind",
                               lapply(split(tertiary_revisions_modified,
tertiary_revisions_modified$.imp),
                                      add_interaction))

# Convert back to mids object
tertiary_revisions_modified <- as.mids(tertiary_revisions_modified)

# Fit the logistic regression model with the interaction term
```

```
model <- with(tertiary_revisions_modified, glm(Mort90days ~ OffPeakDriveDistanceMiles +
Interaction,
                                  family = binomial(link = "logit")))

# Pool the results
pooled_results <- pool(model)

# Summarize pooled results
summary_pooled <- summary(pooled_results, conf.int = TRUE)

# Extract the p-value for the interaction term
box_tidwell_p <- summary_pooled[summary_pooled$term == "Interaction", "p.value"]

# Print the p-value
print(box_tidwell_p)

#0.989



#Exposure 3 - PeakDriveTime



library("lme4")

# Fit logistic regression on imputed datasets include ProvCode in fixed effects to account for
clustering
m3.mi <- with(tertiary_revisions, glm(Mort90days ~ PeakDriveTime + IMD_score +
HFRS_Band +
                              sex + age_of_patient + infection + TV12mo + CV12mo + FinY +
ProvCode,
                            family = "binomial"))


print(m3.mi)


# Pool results across imputed datasets
pooled_results <- pool(m3.mi)

# Summarize pooled results with confidence intervals
summary_pooled <- summary(pooled_results, conf.int = TRUE)

# Add Odds Ratios to the summary
summary_pooled$OR <- exp(summary_pooled$estimate)
summary_pooled$Lower_CI <- exp(summary_pooled$`2.5 %`)
```

```
summary_pooled$Upper_CI <- exp(summary_pooled$`97.5 %`)

# Display the final table with Odds Ratios and Confidence Intervals
print(summary_pooled)

#check for evidence of multicollinearity?

library(car)

# Use the first imputed dataset for the VIF calculation
complete_data <- complete(tertiary_revisions, 1)

# Fit a logistic regression model on the complete dataset
vif_model <- glm(Mort90days ~ PeakDriveTime + IMD_score + HFRS_Band +
                  sex + age_of_patient + infection + TV12mo + CV12mo + FinY + ProvCode,
                data = complete_data, family = "binomial")

# Calculate VIF
vif_values <- vif(vif_model)
print(vif_values)


#No evidence of multi-collinearity

#Is there evidence of non linearity?

# Custom function to add log-transformed variable and interaction term
add_interaction <- function(data) {
  data$Log_PeakDriveTime <- log(data$PeakDriveTime)  # Add log-transformed variable
  data$Interaction <- data$PeakDriveTime * data$Log_PeakDriveTime  # Add interaction
term
  return(data)
}

# Extract the long-format data including the original data
tertiary_revisions_modified <- complete(tertiary_revisions, action = "long", include = TRUE)

# Apply the transformation to each imputed dataset
tertiary_revisions_modified <- do.call("rbind",
                                 lapply(split(tertiary_revisions_modified,
tertiary_revisions_modified$.imp),
                                     add_interaction))

# Convert back to mids object
tertiary_revisions_modified <- as.mids(tertiary_revisions_modified)

# Fit the logistic regression model with the interaction term
```

```
model <- with(tertiary_revisions_modified, glm(Mort90days ~ PeakDriveTime + Interaction,
                                     family = binomial(link = "logit")))

# Pool the results
pooled_results <- pool(model)

# Summarize pooled results
summary_pooled <- summary(pooled_results, conf.int = TRUE)

# Extract the p-value for the interaction term
box_tidwell_p <- summary_pooled[summary_pooled$term == "Interaction", "p.value"]

# Print the p-value
print(box_tidwell_p)


# P avlue 0.78

####Secondary outcome prolonged LOS ####

tertiary_revisions <- complete(tertiary_revisions, "long", inc = TRUE)

tertiary_revisions$Long_Los <- ifelse(tertiary_revisions$Spell_Los > 5, 1, 0)

tertiary_revisions <- as.mids(tertiary_revisions)

#Exposure 1 - Distance Miles

library("lme4")

# Fit logistic regression on imputed datasets include ProvCode in fixed effects to account for
clustering
m3.mi <- with(tertiary_revisions, glm(Long_Los ~ DistanceMiles + IMD_score + HFRS_Band +
                              sex + age_of_patient + infection + TV12mo + CV12mo + FinY +
ProvCode,
                            family = "binomial"))


print(m3.mi)


# Pool results across imputed datasets
pooled_results <- pool(m3.mi)

# Summarize pooled results with confidence intervals
summary_pooled <- summary(pooled_results, conf.int = TRUE)
```

```
# Add Odds Ratios to the summary
summary_pooled$OR <- exp(summary_pooled$estimate)
summary_pooled$Lower_CI <- exp(summary_pooled$`2.5 %`)
summary_pooled$Upper_CI <- exp(summary_pooled$`97.5 %`)

# Display the final table with Odds Ratios and Confidence Intervals
print(summary_pooled)

#check for evidence of multicollinearity?

library(car)

# Use the first imputed dataset for the VIF calculation
complete_data <- complete(tertiary_revisions, 1)

# Fit a logistic regression model on the complete dataset
vif_model <- glm(Long_Los ~ DistanceMiles + IMD_score + HFRS_Band +
                  sex + age_of_patient + infection + TV12mo + CV12mo + FinY + ProvCode,
                data = complete_data, family = "binomial")

# Calculate VIF
vif_values <- vif(vif_model)
print(vif_values)


#No evidence of multi-collinearity

#Is there evidence of non linearity?

# Custom function to add log-transformed variable and interaction term
add_interaction <- function(data) {
  data$Log_DistanceMiles <- log(data$DistanceMiles)  # Add log-transformed variable
  data$Interaction <- data$DistanceMiles * data$Log_DistanceMiles  # Add interaction term
  return(data)
}

# Extract the long-format data including the original data
tertiary_revisions_modified <- complete(tertiary_revisions, action = "long", include = TRUE)

# Apply the transformation to each imputed dataset
tertiary_revisions_modified <- do.call("rbind",
                                     lapply(split(tertiary_revisions_modified,
tertiary_revisions_modified$.imp),
                                            add_interaction))

# Convert back to mids object
tertiary_revisions_modified <- as.mids(tertiary_revisions_modified)
```

```
# Fit the logistic regression model with the interaction term
model <- with(tertiary_revisions_modified, glm(Long_Los ~ DistanceMiles + Interaction,
                                   family = binomial(link = "logit")))

# Pool the results
pooled_results <- pool(model)

# Summarize pooled results
summary_pooled <- summary(pooled_results, conf.int = TRUE)

# Extract the p-value for the interaction term
box_tidwell_p <- summary_pooled[summary_pooled$term == "Interaction", "p.value"]

# Print the p-value
print(box_tidwell_p)


#P value 0.002 Non linear

# Load the required library
library(splines)

#AIC of non spline model

model <- glm(Long_Los ~ DistanceMiles, data = tertiary_revisions, family = binomial)
summary(model)

#AIC 52853

# Define a function to fit and evaluate spline models with knots based on centiles
evaluate_centile_splines <- function(centiles, data) {
  # Calculate knots based on the specified centiles
  knots <- quantile(data$DistanceMiles, probs = centiles, na.rm = TRUE)

  # Fit a logistic regression model with natural splines using the calculated knots
  model_spline <- glm(Long_Los ~ ns(DistanceMiles, knots = knots),
                family = binomial(link = "logit"),
                data = data)

  # Summarize the model
  summary_model <- summary(model_spline)

  # Extract p-values for the spline terms
  p_values <- summary_model$coefficients[-1, "Pr(>|z|)"]  # Exclude the intercept

  # Print the results
```

```
  cat("\nResults for centiles", centiles, ":\n")
  print(p_values)

  # Return the model and calculated knots for further inspection if needed
  return(list(model = model_spline, p_values = p_values, knots = knots))
}

# Example centile configurations for 3, 4, and 5 knots
centiles_3_knots <- c(0.05, 0.50, 0.95)  # 5th, 50th, and 95th percentiles
centiles_4_knots <- c(0.05, 0.35, 0.65, 0.95)  # Custom centiles for 4 knots
centiles_5_knots <- c(0.05, 0.25, 0.50, 0.75, 0.95)  # 5 knots centiles

# Evaluate models with centile-based knots using your dataset
results_3_knots <- evaluate_centile_splines(centiles = centiles_3_knots, data =
tertiary_revisions)
results_4_knots <- evaluate_centile_splines(centiles = centiles_4_knots, data =
tertiary_revisions)
results_5_knots <- evaluate_centile_splines(centiles = centiles_5_knots, data =
tertiary_revisions)

# Compare models with centile-based knots
cat("\nComparing models with different centile-based knots:\n")
anova(results_3_knots$model, results_4_knots$model, results_5_knots$model, test =
"Chisq")

# Print the calculated knot locations for each model
cat("\nKnot locations for 3 knots:\n")
print(results_3_knots$knots)
cat("\nKnot locations for 4 knots:\n")
print(results_4_knots$knots)
cat("\nKnot locations for 5 knots:\n")
print(results_5_knots$knots)

#52769, model with four knots best fit and improved fit from original linear model

#Run spline model with adjusted data excluding missing data
library(splines)
# For example, let's say you want 3 knots at specific percentiles
knots <- quantile(tertiary_revisions$DistanceMiles, probs = c(0.05, 0.35, 0.65, 0.95), na.rm =
TRUE)
print(knots)

spline_terms <- ns(tertiary_revisions$DistanceMiles, knots = knots)
```

```
model_with_custom_splines <- glm(Long_Los ~ ns(DistanceMiles, knots = knots) +
HFRS_Band + IMD_score +
                                  sex + age_of_patient + infection + TV12mo + CV12mo + FinY + ProvCode,
                                family = "binomial", data = tertiary_revisions)


summary(model_with_custom_splines)

#Generate a sequence of mean unit values for predicting

DistanceMiles_range <- seq(min(tertiary_revisions$DistanceMiles),
max(tertiary_revisions$DistanceMiles), length.out = 100)

new_data <- expand.grid(
  DistanceMiles = DistanceMiles_range,
  sex = levels(tertiary_revisions$sex),  # Ensure it takes all factor levels
  age_of_patient = mean(tertiary_revisions$age_of_patient, na.rm = TRUE),
  HFRS_Band = levels(tertiary_revisions$HFRS_Band),  # Ensuring correct factor levels
  IMD_score = mean(tertiary_revisions$IMD_score, na.rm = TRUE),
  FinY = levels(tertiary_revisions$FinY),  # Ensuring correct factor levels
  CV12mo = mean(tertiary_revisions$CV12mo, na.rm = TRUE),
  TV12mo = mean(tertiary_revisions$TV12mo, na.rm = TRUE),
  ProvCode = levels(tertiary_revisions$ProvCode),  # Ensuring correct factor levels
  infection = levels(tertiary_revisions$infection)  # Ensuring correct factor levels
)

# Create a new dataset with a range of distances and miles and all other predictor variables
new_data <- expand.grid(DistanceMiles = DistanceMiles_range,
                        sex = unique(tertiary_revisions$sex),
                        age_of_patient = mean(tertiary_revisions$age_of_patient),
                        HFRS_Band = unique(tertiary_revisions$HFRS_Band),
                        IMD_score = mean(tertiary_revisions$IMD_score),
                        FinY = unique(tertiary_revisions$FinY),
                        CV12mo = mean(tertiary_revisions$CV12mo),
                        TV12mo = mean(tertiary_revisions$TV12mo),
                        infection = unique(tertiary_revisions$infection))


# Align the levels of ProvCode in new_data to match the training data
new_data$ProvCode <- factor(new_data$ProvCode, levels =
levels(tertiary_revisions$ProvCode))

# Align the levels of all relevant categorical variables
new_data$HFRS_Band <- factor(new_data$HFRS_Band, levels =
levels(tertiary_revisions$HFRS_Band))
new_data$sex <- factor(new_data$sex, levels = levels(tertiary_revisions$sex))
new_data$FinY <- factor(new_data$FinY, levels = levels(tertiary_revisions$FinY))
```

```
new_data$infection <- factor(new_data$infection, levels =
levels(tertiary_revisions$infection))

#Factors are consistent with model

levels(new_data$HFRS_Band)
levels(tertiary_revisions$HFRS_Band)

levels(new_data$sex)
levels(tertiary_revisions$sex)

levels(new_data$FinY)
levels(tertiary_revisions$FinY)

levels(new_data$ProvCode)
levels(tertiary_revisions$ProvCode)

levels(new_data$infection)
levels(tertiary_revisions$infection)

# Check levels of ProvCode in both datasets
setdiff(levels(new_data$ProvCode), levels(tertiary_revisions$ProvCode))  # Levels in
new_data but not in tertiary_revisions
setdiff(levels(tertiary_revisions$ProvCode), levels(new_data$ProvCode))  # Levels in
tertiary_revisions but not in new_data

new_data$ProvCode <- droplevels(new_data$ProvCode)
# Check for missing values in factor variables
sum(is.na(new_data$ProvCode))  # Number of missing values in ProvCode

# Ensure that ProvCode is a factor
new_data$ProvCode <- factor(new_data$ProvCode, levels =
levels(tertiary_revisions$ProvCode))

# Now try the prediction again
predicted_probs <- predict(model_with_custom_splines, newdata = new_data, type =
"response")




# Combine mean_unit_range and predicted_probs into a data frame
plot_data <- data.frame(DistanceMiles = DistanceMiles_range, predicted_prob =
predicted_probs)

#Calculate 95% confidence intervals
```

```
# Obtain predicted values and standard errors for the new data
predictions <- predict(model_with_custom_splines, newdata = new_data, type = "link",
se.fit = TRUE)

# Calculate the confidence intervals for the log-odds scale (link scale)
# Use a 95% confidence level (z-value = 1.96 for a 95% CI)
z_value <- 1.96
log_odds_lower <- predictions$fit - z_value * predictions$se.fit
log_odds_upper <- predictions$fit + z_value * predictions$se.fit

# Convert the log-odds confidence intervals to probabilities
# First, apply the inverse link function (logistic function) to the log-odds
lower_prob <- plogis(log_odds_lower)
upper_prob <- plogis(log_odds_upper)

# Combine the predicted probabilities and their confidence intervals into a data frame
plot_data <- data.frame(
  DistanceMiles = new_data$DistanceMiles,
  predicted_prob = plogis(predictions$fit),  # Logistic transformation of the link
  ci_lower = lower_prob,
  ci_upper = upper_prob
)




library(ggplot2)
# Plot the spline curve with confidence intervals
ggplot(plot_data, aes(x = DistanceMiles)) +
  geom_line(aes(y = predicted_prob), color = "blue", size = 1) +
  geom_ribbon(aes(ymin = ci_lower, ymax = ci_upper), fill = "blue", alpha = 0.2) +
  labs(x = "Distance (Miles)", y = "Predicted Probability Readmission at 30 days") +
  theme_minimal()

library(dplyr)

# Group by mean_unit and calculate mean predicted_prob and corresponding confidence
intervals
mean_data <- plot_data %>%
  group_by(DistanceMiles) %>%
  summarise(
    mean_predicted_prob = mean(predicted_prob, na.rm = TRUE),
    mean_ci_lower = mean(ci_lower, na.rm = TRUE),
    mean_ci_upper = mean(ci_upper, na.rm = TRUE)
  )
```

```
# Define specific breaks (e.g., 25, 50, 75, ..., up to the maximum)
breaks_seq <- seq(0, max(mean_data$DistanceMiles, na.rm = TRUE), by = 5)

library(ggplot2)
# Plot with specified increments on x-axis
ggplot(mean_data, aes(x = DistanceMiles, y = mean_predicted_prob)) +
  geom_point() +  # Add points for mean_predicted_prob
  geom_line() +   # Connect points with a line
  geom_ribbon(aes(ymin = mean_ci_lower, ymax = mean_ci_upper), fill = "blue", alpha =
0.2) +  # Add ribbon for confidence intervals
  labs(x = "Travel Distance (Miles)", y = "Mean Predicted Probability for Prolonged LOS", title
= "Spline curve predicted probability of prolonged LOS by patient travel distance") +
  scale_x_continuous(limits = c(0, max(mean_data$DistanceMiles, na.rm = TRUE)), breaks =
breaks_seq) +
  theme_minimal() +
  theme(
    axis.title.x = element_text(size = 14),  # Increase x-axis title font size
    axis.title.y = element_text(size = 14),  # Increase y-axis title font size
    axis.text.x = element_text(size = 12),   # Increase x-axis tick label font size
    axis.text.y = element_text(size = 12),   # Increase y-axis tick label font size
    plot.title = element_text(size = 16, hjust = 0.5)  # Increase plot title font size and center it
  )



#Exposure 2 - OffPeakDriveDistanceMiles



library("lme4")

# Fit logistic regression on imputed datasets include ProvCode in fixed effects to account for
clustering
m3.mi <- with(tertiary_revisions, glm(Long_Los ~ OffPeakDriveDistanceMiles + IMD_score +
HFRS_Band +
                                      sex + age_of_patient + infection + TV12mo + CV12mo + FinY +
ProvCode,
                                    family = "binomial"))


print(m3.mi)


# Pool results across imputed datasets
pooled_results <- pool(m3.mi)
```

```
# Summarize pooled results with confidence intervals
summary_pooled <- summary(pooled_results, conf.int = TRUE)

# Add Odds Ratios to the summary
summary_pooled$OR <- exp(summary_pooled$estimate)
summary_pooled$Lower_CI <- exp(summary_pooled$`2.5 %`)
summary_pooled$Upper_CI <- exp(summary_pooled$`97.5 %`)

# Display the final table with Odds Ratios and Confidence Intervals
print(summary_pooled)

#check for evidence of multicollinearity?

library(car)

# Use the first imputed dataset for the VIF calculation
complete_data <- complete(tertiary_revisions, 1)

# Fit a logistic regression model on the complete dataset
vif_model <- glm(Read30days ~ DistanceMiles + IMD_score + HFRS_Band +
                 sex + age_of_patient + infection + TV12mo + CV12mo + FinY + ProvCode,
                data = complete_data, family = "binomial")

# Calculate VIF
vif_values <- vif(vif_model)
print(vif_values)


#No evidence of multi-collinearity

#Is there evidence of non linearity?

# Custom function to add log-transformed variable and interaction term
add_interaction <- function(data) {
  data$Log_OffPeakDriveDistanceMiles <- log(data$OffPeakDriveDistanceMiles)  # Add log-
transformed variable
  data$Interaction <- data$OffPeakDriveDistanceMiles *
data$Log_OffPeakDriveDistanceMiles  # Add interaction term
  return(data)
}

# Extract the long-format data including the original data
tertiary_revisions_modified <- complete(tertiary_revisions, action = "long", include = TRUE)

# Apply the transformation to each imputed dataset
tertiary_revisions_modified <- do.call("rbind",
```

```
                              lapply(split(tertiary_revisions_modified,
tertiary_revisions_modified$.imp),
                                   add_interaction))

# Convert back to mids object
tertiary_revisions_modified <- as.mids(tertiary_revisions_modified)

# Fit the logistic regression model with the interaction term
model <- with(tertiary_revisions_modified, glm(Long_Los ~ OffPeakDriveDistanceMiles +
Interaction,
                                    family = binomial(link = "logit")))

# Pool the results
pooled_results <- pool(model)

# Summarize pooled results
summary_pooled <- summary(pooled_results, conf.int = TRUE)

# Extract the p-value for the interaction term
box_tidwell_p <- summary_pooled[summary_pooled$term == "Interaction", "p.value"]

# Print the p-value
print(box_tidwell_p)

#0.003

#AIC of non spline model

model <- glm(Long_Los ~ OffPeakDriveDistanceMiles, data = tertiary_revisions, family =
binomial)
summary(model)

#AIC 52853

# Define a function to fit and evaluate spline models with knots based on centiles
evaluate_centile_splines <- function(centiles, data) {
  # Calculate knots based on the specified centiles
  knots <- quantile(data$OffPeakDriveDistanceMiles, probs = centiles, na.rm = TRUE)

  # Fit a logistic regression model with natural splines using the calculated knots
  model_spline <- glm(Long_Los ~ ns(OffPeakDriveDistanceMiles, knots = knots),
                family = binomial(link = "logit"),
                data = data)

  # Summarize the model
  summary_model <- summary(model_spline)
```

```
  # Extract p-values for the spline terms
  p_values <- summary_model$coefficients[-1, "Pr(>|z|)"]  # Exclude the intercept

  # Print the results
  cat("\nResults for centiles", centiles, ":\n")
  print(p_values)

  # Return the model and calculated knots for further inspection if needed
  return(list(model = model_spline, p_values = p_values, knots = knots))
}

# Example centile configurations for 3, 4, and 5 knots
centiles_3_knots <- c(0.05, 0.50, 0.95)  # 5th, 50th, and 95th percentiles
centiles_4_knots <- c(0.05, 0.35, 0.65, 0.95)  # Custom centiles for 4 knots
centiles_5_knots <- c(0.05, 0.25, 0.50, 0.75, 0.95)  # 5 knots centiles

# Evaluate models with centile-based knots using your dataset
results_3_knots <- evaluate_centile_splines(centiles = centiles_3_knots, data =
tertiary_revisions)
results_4_knots <- evaluate_centile_splines(centiles = centiles_4_knots, data =
tertiary_revisions)
results_5_knots <- evaluate_centile_splines(centiles = centiles_5_knots, data =
tertiary_revisions)

# Compare models with centile-based knots
cat("\nComparing models with different centile-based knots:\n")
anova(results_3_knots$model, results_4_knots$model, results_5_knots$model, test =
"Chisq")

# Print the calculated knot locations for each model
cat("\nKnot locations for 3 knots:\n")
print(results_3_knots$knots)
cat("\nKnot locations for 4 knots:\n")
print(results_4_knots$knots)
cat("\nKnot locations for 5 knots:\n")
print(results_5_knots$knots)

#52718, model with four knots best fit and significant spline terms

#Run spline model with adjusted data excluding missing data
library(splines)
# For example, let's say you want 3 knots at specific percentiles
knots <- quantile(tertiary_revisions$OffPeakDriveDistanceMiles, probs = c(0.05, 0.35, 0.65,
0.95), na.rm = TRUE)
print(knots)

spline_terms <- ns(tertiary_revisions$OffPeakDriveDistanceMiles, knots = knots)
```

```
model_with_custom_splines <- glm(Long_Los ~ ns(OffPeakDriveDistanceMiles, knots =
knots) + HFRS_Band + IMD_score +
                                 sex + age_of_patient + infection + TV12mo + CV12mo + FinY + ProvCode,
                               family = "binomial", data = tertiary_revisions)


summary(model_with_custom_splines)

#Generate a sequence of mean unit values for predicting

DistanceMiles_range <- seq(min(tertiary_revisions$OffPeakDriveDistanceMiles),
max(tertiary_revisions$OffPeakDriveDistanceMiles), length.out = 100)

new_data <- expand.grid(
  OffPeakDriveDistanceMiles = DistanceMiles_range,
  sex = levels(tertiary_revisions$sex),  # Ensure it takes all factor levels
  age_of_patient = mean(tertiary_revisions$age_of_patient, na.rm = TRUE),
  HFRS_Band = levels(tertiary_revisions$HFRS_Band),  # Ensuring correct factor levels
  IMD_score = mean(tertiary_revisions$IMD_score, na.rm = TRUE),
  FinY = levels(tertiary_revisions$FinY),  # Ensuring correct factor levels
  CV12mo = mean(tertiary_revisions$CV12mo, na.rm = TRUE),
  TV12mo = mean(tertiary_revisions$TV12mo, na.rm = TRUE),
  ProvCode = levels(tertiary_revisions$ProvCode),  # Ensuring correct factor levels
  infection = levels(tertiary_revisions$infection)  # Ensuring correct factor levels
)

# Create a new dataset with a range of distances and miles and all other predictor variables
new_data <- expand.grid(DistanceMiles = DistanceMiles_range,
                        sex = unique(tertiary_revisions$sex),
                        age_of_patient = mean(tertiary_revisions$age_of_patient),
                        HFRS_Band = unique(tertiary_revisions$HFRS_Band),
                        IMD_score = mean(tertiary_revisions$IMD_score),
                        FinY = unique(tertiary_revisions$FinY),
                        CV12mo = mean(tertiary_revisions$CV12mo),
                        TV12mo = mean(tertiary_revisions$TV12mo),
                        infection = unique(tertiary_revisions$infection))


# Align the levels of ProvCode in new_data to match the training data
new_data$ProvCode <- factor(new_data$ProvCode, levels =
levels(tertiary_revisions$ProvCode))

# Align the levels of all relevant categorical variables
```

```
new_data$HFRS_Band <- factor(new_data$HFRS_Band, levels =
levels(tertiary_revisions$HFRS_Band))
new_data$sex <- factor(new_data$sex, levels = levels(tertiary_revisions$sex))
new_data$FinY <- factor(new_data$FinY, levels = levels(tertiary_revisions$FinY))
new_data$infection <- factor(new_data$infection, levels =
levels(tertiary_revisions$infection))

#Factors are consistent with model

levels(new_data$HFRS_Band)
levels(tertiary_revisions$HFRS_Band)

levels(new_data$sex)
levels(tertiary_revisions$sex)

levels(new_data$FinY)
levels(tertiary_revisions$FinY)

levels(new_data$ProvCode)
levels(tertiary_revisions$ProvCode)

levels(new_data$infection)
levels(tertiary_revisions$infection)

# Check levels of ProvCode in both datasets
setdiff(levels(new_data$ProvCode), levels(tertiary_revisions$ProvCode))  # Levels in
new_data but not in tertiary_revisions
setdiff(levels(tertiary_revisions$ProvCode), levels(new_data$ProvCode))  # Levels in
tertiary_revisions but not in new_data

new_data$ProvCode <- droplevels(new_data$ProvCode)
# Check for missing values in factor variables
sum(is.na(new_data$ProvCode))  # Number of missing values in ProvCode

# Ensure that ProvCode is a factor
new_data$ProvCode <- factor(new_data$ProvCode, levels =
levels(tertiary_revisions$ProvCode))

# Now try the prediction again
predicted_probs <- predict(model_with_custom_splines, newdata = new_data, type =
"response")




# Combine mean_unit_range and predicted_probs into a data frame
```

```
plot_data <- data.frame(OffPeakDriveDistanceMiles = DistanceMiles_range, predicted_prob
= predicted_probs)

#Calculate 95% confidence intervals

# Obtain predicted values and standard errors for the new data
predictions <- predict(model_with_custom_splines, newdata = new_data, type = "link",
se.fit = TRUE)

# Calculate the confidence intervals for the log-odds scale (link scale)
# Use a 95% confidence level (z-value = 1.96 for a 95% CI)
z_value <- 1.96
log_odds_lower <- predictions$fit - z_value * predictions$se.fit
log_odds_upper <- predictions$fit + z_value * predictions$se.fit

# Convert the log-odds confidence intervals to probabilities
# First, apply the inverse link function (logistic function) to the log-odds
lower_prob <- plogis(log_odds_lower)
upper_prob <- plogis(log_odds_upper)

# Combine the predicted probabilities and their confidence intervals into a data frame
plot_data <- data.frame(
  DistanceMiles = new_data$OffPeakDriveDistanceMiles,
  predicted_prob = plogis(predictions$fit),  # Logistic transformation of the link
  ci_lower = lower_prob,
  ci_upper = upper_prob
)




library(ggplot2)
# Plot the spline curve with confidence intervals
ggplot(plot_data, aes(x = DistanceMiles)) +
  geom_line(aes(y = predicted_prob), color = "blue", size = 1) +
  geom_ribbon(aes(ymin = ci_lower, ymax = ci_upper), fill = "blue", alpha = 0.2) +
  labs(x = "Distance (Miles)", y = "Predicted Probability Readmission at 30 days") +
  theme_minimal()

library(dplyr)

# Group by mean_unit and calculate mean predicted_prob and corresponding confidence
intervals
mean_data <- plot_data %>%
  group_by(DistanceMiles) %>%
  summarise(
    mean_predicted_prob = mean(predicted_prob, na.rm = TRUE),
```

```
    mean_ci_lower = mean(ci_lower, na.rm = TRUE),
    mean_ci_upper = mean(ci_upper, na.rm = TRUE)
  )


# Define specific breaks (e.g., 25, 50, 75, ..., up to the maximum)
breaks_seq <- seq(0, max(mean_data$DistanceMiles, na.rm = TRUE), by = 5)

library(ggplot2)
# Plot with specified increments on x-axis
ggplot(mean_data, aes(x = DistanceMiles, y = mean_predicted_prob)) +
  geom_point() +  # Add points for mean_predicted_prob
  geom_line() +   # Connect points with a line
  geom_ribbon(aes(ymin = mean_ci_lower, ymax = mean_ci_upper), fill = "blue", alpha =
0.2) +  # Add ribbon for confidence intervals
  labs(x = "Off Peak Drive Distance Miles", y = "Mean Predicted Probability for Prolonged
LOS", title = "Spline curve predicted probability of prolonged LOS by patient driving
distance") +
  scale_x_continuous(limits = c(0, max(mean_data$DistanceMiles, na.rm = TRUE)), breaks =
breaks_seq) +
  theme_minimal() +
  theme(
    axis.title.x = element_text(size = 14),  # Increase x-axis title font size
    axis.title.y = element_text(size = 14),  # Increase y-axis title font size
    axis.text.x = element_text(size = 12),   # Increase x-axis tick label font size
    axis.text.y = element_text(size = 12),   # Increase y-axis tick label font size
    plot.title = element_text(size = 16, hjust = 0.5)  # Increase plot title font size and center it
  )



#Exposure 3 - PeakDriveTime



library("lme4")

# Fit logistic regression on imputed datasets include ProvCode in fixed effects to account for
clustering
m3.mi <- with(tertiary_revisions, glm(Long_Los ~ PeakDriveTime + IMD_score + HFRS_Band
+
                                      sex + age_of_patient + infection + TV12mo + CV12mo + FinY +
ProvCode,
                                    family = "binomial"))
```

```
print(m3.mi)


# Pool results across imputed datasets
pooled_results <- pool(m3.mi)

# Summarize pooled results with confidence intervals
summary_pooled <- summary(pooled_results, conf.int = TRUE)

# Add Odds Ratios to the summary
summary_pooled$OR <- exp(summary_pooled$estimate)
summary_pooled$Lower_CI <- exp(summary_pooled$`2.5 %`)
summary_pooled$Upper_CI <- exp(summary_pooled$`97.5 %`)

# Display the final table with Odds Ratios and Confidence Intervals
print(summary_pooled)

#check for evidence of multicollinearity?

library(car)

# Use the first imputed dataset for the VIF calculation
complete_data <- complete(tertiary_revisions, 1)

# Fit a logistic regression model on the complete dataset
vif_model <- glm(Read30days ~ DistanceMiles + IMD_score + HFRS_Band +
                 sex + age_of_patient + infection + TV12mo + CV12mo + FinY + ProvCode,
                data = complete_data, family = "binomial")

# Calculate VIF
vif_values <- vif(vif_model)
print(vif_values)


#Is there evidence of non linearity?

# Custom function to add log-transformed variable and interaction term
add_interaction <- function(data) {
  data$Log_PeakDriveTime <- log(data$PeakDriveTime)  # Add log-transformed variable
  data$Interaction <- data$PeakDriveTime * data$Log_PeakDriveTime  # Add interaction
term
  return(data)
}

# Extract the long-format data including the original data
tertiary_revisions_modified <- complete(tertiary_revisions, action = "long", include = TRUE)
```

```
# Apply the transformation to each imputed dataset
tertiary_revisions_modified <- do.call("rbind",
                                       lapply(split(tertiary_revisions_modified,
tertiary_revisions_modified$.imp),
                                              add_interaction))

# Convert back to mids object
tertiary_revisions_modified <- as.mids(tertiary_revisions_modified)

# Fit the logistic regression model with the interaction term
model <- with(tertiary_revisions_modified, glm(Long_Los ~ PeakDriveTime + Interaction,
                                               family = binomial(link = "logit")))

# Pool the results
pooled_results <- pool(model)

# Summarize pooled results
summary_pooled <- summary(pooled_results, conf.int = TRUE)

# Extract the p-value for the interaction term
box_tidwell_p <- summary_pooled[summary_pooled$term == "Interaction", "p.value"]

# Print the p-value
print(box_tidwell_p)

#P value 0.000916

#AIC of non spline model

model <- glm(Long_Los ~ PeakDriveTime, data = tertiary_revisions, family = binomial)
summary(model)

#AIC 52843

# Define a function to fit and evaluate spline models with knots based on centiles
evaluate_centile_splines <- function(centiles, data) {
  # Calculate knots based on the specified centiles
  knots <- quantile(data$PeakDriveTime, probs = centiles, na.rm = TRUE)

  # Fit a logistic regression model with natural splines using the calculated knots
  model_spline <- glm(Long_Los ~ ns(PeakDriveTime, knots = knots),
                      family = binomial(link = "logit"),
                      data = data)

  # Summarize the model
  summary_model <- summary(model_spline)
```

```
  # Extract p-values for the spline terms
  p_values <- summary_model$coefficients[-1, "Pr(>|z|)"]  # Exclude the intercept

  # Print the results
  cat("\nResults for centiles", centiles, ":\n")
  print(p_values)

  # Return the model and calculated knots for further inspection if needed
  return(list(model = model_spline, p_values = p_values, knots = knots))
}

# Example centile configurations for 3, 4, and 5 knots
centiles_3_knots <- c(0.05, 0.50, 0.95)  # 5th, 50th, and 95th percentiles
centiles_4_knots <- c(0.05, 0.35, 0.65, 0.95)  # Custom centiles for 4 knots
centiles_5_knots <- c(0.05, 0.25, 0.50, 0.75, 0.95)  # 5 knots centiles

# Evaluate models with centile-based knots using your dataset
results_3_knots <- evaluate_centile_splines(centiles = centiles_3_knots, data =
tertiary_revisions)
results_4_knots <- evaluate_centile_splines(centiles = centiles_4_knots, data =
tertiary_revisions)
results_5_knots <- evaluate_centile_splines(centiles = centiles_5_knots, data =
tertiary_revisions)

# Compare models with centile-based knots
cat("\nComparing models with different centile-based knots:\n")
anova(results_3_knots$model, results_4_knots$model, results_5_knots$model, test =
"Chisq")

# Print the calculated knot locations for each model
cat("\nKnot locations for 3 knots:\n")
print(results_3_knots$knots)
cat("\nKnot locations for 4 knots:\n")
print(results_4_knots$knots)
cat("\nKnot locations for 5 knots:\n")
print(results_5_knots$knots)

#52715, model with four knots best fit and significant spline terms and most parsimonious

#Run spline model with adjusted data excluding missing data
library(splines)
# For example, let's say you want 3 knots at specific percentiles
knots <- quantile(tertiary_revisions$PeakDriveTime, probs = c(0.05, 0.35, 0.65, 0.95), na.rm
= TRUE)
print(knots)

spline_terms <- ns(tertiary_revisions$PeakDriveTime, knots = knots)
```

```
model_with_custom_splines <- glm(Long_Los ~ ns(PeakDriveTime, knots = knots) +
HFRS_Band + IMD_score +
                        sex + age_of_patient + infection + TV12mo + CV12mo + FinY + ProvCode,
                      family = "binomial", data = tertiary_revisions)


summary(model_with_custom_splines)

#Generate a sequence of mean unit values for predicting

DistanceMiles_range <- seq(min(tertiary_revisions$PeakDriveTime),
max(tertiary_revisions$PeakDriveTime), length.out = 100)

new_data <- expand.grid(
  PeakDriveTime = DistanceMiles_range,
  sex = levels(tertiary_revisions$sex),  # Ensure it takes all factor levels
  age_of_patient = mean(tertiary_revisions$age_of_patient, na.rm = TRUE),
  HFRS_Band = levels(tertiary_revisions$HFRS_Band),  # Ensuring correct factor levels
  IMD_score = mean(tertiary_revisions$IMD_score, na.rm = TRUE),
  FinY = levels(tertiary_revisions$FinY),  # Ensuring correct factor levels
  CV12mo = mean(tertiary_revisions$CV12mo, na.rm = TRUE),
  TV12mo = mean(tertiary_revisions$TV12mo, na.rm = TRUE),
  ProvCode = levels(tertiary_revisions$ProvCode),  # Ensuring correct factor levels
  infection = levels(tertiary_revisions$infection)  # Ensuring correct factor levels
)


# Align the levels of ProvCode in new_data to match the training data
new_data$ProvCode <- factor(new_data$ProvCode, levels =
levels(tertiary_revisions$ProvCode))

# Align the levels of all relevant categorical variables
new_data$HFRS_Band <- factor(new_data$HFRS_Band, levels =
levels(tertiary_revisions$HFRS_Band))
new_data$sex <- factor(new_data$sex, levels = levels(tertiary_revisions$sex))
new_data$FinY <- factor(new_data$FinY, levels = levels(tertiary_revisions$FinY))
new_data$infection <- factor(new_data$infection, levels =
levels(tertiary_revisions$infection))

#Factors are consistent with model

levels(new_data$HFRS_Band)
levels(tertiary_revisions$HFRS_Band)

levels(new_data$sex)
```

```
levels(tertiary_revisions$sex)

levels(new_data$FinY)
levels(tertiary_revisions$FinY)

levels(new_data$ProvCode)
levels(tertiary_revisions$ProvCode)

levels(new_data$infection)
levels(tertiary_revisions$infection)

# Check levels of ProvCode in both datasets
setdiff(levels(new_data$ProvCode), levels(tertiary_revisions$ProvCode))  # Levels in new_data but not in tertiary_revisions
setdiff(levels(tertiary_revisions$ProvCode), levels(new_data$ProvCode))  # Levels in tertiary_revisions but not in new_data

new_data$ProvCode <- droplevels(new_data$ProvCode)
# Check for missing values in factor variables
sum(is.na(new_data$ProvCode))  # Number of missing values in ProvCode

# Ensure that ProvCode is a factor
new_data$ProvCode <- factor(new_data$ProvCode, levels = levels(tertiary_revisions$ProvCode))

# Now try the prediction again
predicted_probs <- predict(model_with_custom_splines, newdata = new_data, type = "response")




# Combine mean_unit_range and predicted_probs into a data frame
plot_data <- data.frame(PeakDriveTime = DistanceMiles_range, predicted_prob = predicted_probs)

#Calculate 95% confidence intervals

# Obtain predicted values and standard errors for the new data
predictions <- predict(model_with_custom_splines, newdata = new_data, type = "link", se.fit = TRUE)

# Calculate the confidence intervals for the log-odds scale (link scale)
# Use a 95% confidence level (z-value = 1.96 for a 95% CI)
z_value <- 1.96
log_odds_lower <- predictions$fit - z_value * predictions$se.fit
log_odds_upper <- predictions$fit + z_value * predictions$se.fit
```

```
# Convert the log-odds confidence intervals to probabilities
# First, apply the inverse link function (logistic function) to the log-odds
lower_prob <- plogis(log_odds_lower)
upper_prob <- plogis(log_odds_upper)

# Combine the predicted probabilities and their confidence intervals into a data frame
plot_data <- data.frame(
  DriveTime = new_data$PeakDriveTime,
  predicted_prob = plogis(predictions$fit),  # Logistic transformation of the link
  ci_lower = lower_prob,
  ci_upper = upper_prob
)




library(ggplot2)
# Plot the spline curve with confidence intervals
ggplot(plot_data, aes(x = DriveTime)) +
  geom_line(aes(y = predicted_prob), color = "blue", size = 1) +
  geom_ribbon(aes(ymin = ci_lower, ymax = ci_upper), fill = "blue", alpha = 0.2) +
  labs(x = "Distance (Miles)", y = "Predicted Probability Readmission at 30 days") +
  theme_minimal()

library(dplyr)

# Group by mean_unit and calculate mean predicted_prob and corresponding confidence
intervals
mean_data <- plot_data %>%
  group_by(DriveTime) %>%
  summarise(
    mean_predicted_prob = mean(predicted_prob, na.rm = TRUE),
    mean_ci_lower = mean(ci_lower, na.rm = TRUE),
    mean_ci_upper = mean(ci_upper, na.rm = TRUE)
  )


# Define specific breaks (e.g., 25, 50, 75, ..., up to the maximum)
breaks_seq <- seq(0, max(mean_data$DriveTime, na.rm = TRUE), by = 5)

library(ggplot2)
# Plot with specified increments on x-axis
ggplot(mean_data, aes(x = DriveTime, y = mean_predicted_prob)) +
  geom_point() +  # Add points for mean_predicted_prob
  geom_line() +   # Connect points with a line
```

```
  geom_ribbon(aes(ymin = mean_ci_lower, ymax = mean_ci_upper), fill = "blue", alpha = 
0.2) +  # Add ribbon for confidence intervals
  labs(x = "Peak Drive Times (Minutes)", y = "Mean Predicted Probability for Prolonged LOS", 
title = "Spline curve predicted probability of prolonged LOS by patient driving times") +
  scale_x_continuous(limits = c(0, max(mean_data$DriveTime, na.rm = TRUE)), breaks = 
breaks_seq) +
  theme_minimal() +
  theme(
    axis.title.x = element_text(size = 14),  # Increase x-axis title font size
    axis.title.y = element_text(size = 14),  # Increase y-axis title font size
    axis.text.x = element_text(size = 12),   # Increase x-axis tick label font size
    axis.text.y = element_text(size = 12),   # Increase y-axis tick label font size
    plot.title = element_text(size = 16, hjust = 0.5)  # Increase plot title font size and center it
  )
```

####END####
