# Supplementary material for: What is the impact of longer patient travel distances and times on perioperative outcomes following revision knee replacement: a retrospective observational study using data for England from Hospital Episode Statistics
Source: BMJ Open. 2025 May 6;15(5):e085201. doi: 10.1136/bmjopen-2024-085201 (PMC12056618; doi:10.1136/bmjopen-2024-085201)
Supplement: Supplementary file 6 [file bmjopen-2024-085201.reviewer_comments.pdf]

PEER REVIEW HISTORY

BMJ Open publishes all reviews undertaken for accepted manuscripts. Reviewers are asked to complete a checklist review form and are provided with free text boxes to elaborate on their assessment. These free text comments are reproduced below.

ARTICLE DETAILS

Title (Provisional)

What is the impact of longer patient travel distances and times on perioperative outcomes following revision knee replacement: A retrospective observational study using data for England from Hospital Episode Statistics

Authors

Matthews, Alexander Handel; Evans, Jonathan Peter; Evans, Jonathan Thomas; Lamb, Sarah; Price, Andrew James; Gray, William; Briggs, Tim; Toms, Andrew D

VERSION 1 - REVIEW

**Reviewer**	**1**
**Name**	**Liau, Zi Qiang Glen**
**Affiliation**	**National University Hospital, Orthopaedic Surgery**
**Date**	**06-Apr-2024**
**COI**	**Nil**

Thank you for the engaging topic and findings.

1. Can you please comment on why the choice of quintiles was made? As opposed to analyses across the distances; or in a different division (eg trio / quartile).

2. "Readmission within 30 days" - the results appear that a significant difference may be found, if the distances were evaluated on a continuous basis. Was an analysis performed in such a manner? If not, could this be done to see if there was a significant relationship?

Best regards

**Reviewer**	**2**
**Name**	**Lungu, Daniel**
**Affiliation**	**Scuola Superiore di Studi Universitari e di Perfezionamento Sant'Anna, Health and Management Laboratory (MeS Lab), Institute of Management and Department EMbeDS**
**Date**	**23-Jun-2024**

COI **None**

I had the opportunity to read your manuscript investigating the association between travel time/distance and the perioperative outcomes after complex revision knee surgery.

While presenting everything clearly, and this being a methodologically robust study, I am not able to see the scientific interest. It is for sure a key result for policy makers, not for the rest of the scientific community. This is because the research question is not adequately grounded.

Why should travel distances/time have an impact on postoperative outcomes? Is there an assumption? A theory? A hypothesis? I cannot find one. Moreover, I am not surprised by your findings of no difference, since you are dealing with an elective intervention. The (de)centralisation of interventions is widely debated in international literature, which I recommend reading.

Just as a final example, I might want to investigate if the size (in sqm) of these patients' homes has an effect on their perioperative outcomes. While intellectually stimulating, I would not find any scientific element to ground my study. I think you are experiencing a very similar issue.

VERSION 1 - AUTHOR RESPONSE

Reviewer: 1 Comments

Dr. Zi Qiang Glen Liau, National University Hospital

Comments to the Author:

Thank you for the engaging topic and findings.

1. Can you please comment on why the choice of quintiles was made? As opposed to analyses across the distances; or in a different division (eg trio / quartile).

[We followed an a priori methodology based on a previous study investigating travel times and perioperative outcomes for patients undergoing thoracic surgery. Travel times in this paper were divided into quintiles. I have edited our manuscript to state this this was chosen a priori.]

2. "Readmission within 30 days" - the results appear that a significant difference may be found, if the distances were evaluated on a continuous basis. Was an analysis performed in such a manner? If not, could this be done to see if there was a significant relationship?
[I have included supplementary material exploring this relationship in detail. In summary there is no statistically significant relationship between distance or time (modelling as continuous) and 30 day readmission rates]

Reviewer 2: Comments

I had the opportunity to read your manuscript investigating the association between travel time/distance and the perioperative outcomes after complex revision knee surgery.
While presenting everything clearly, and this being a methodologically robust study, I am not able to see the scientific interest. It is for sure a key result for policy makers, not for the rest of the scientific community. This is because the research question is not adequately grounded.
Why should travel distances/time have an impact on postoperative outcomes? Is there an assumption? A theory? A hypothesis? I cannot find one. Moreover, I am not surprised by your findings of no difference, since you are dealing with an elective intervention. The (de)centralisation of interventions is widely debated in international literature, which I recommend reading.
Just as a final example, I might want to investigate if the size (in sqm) of these patients' homes has an effect on their perioperative outcomes. While intellectually stimulating, I would not find any scientific element to ground my study. I think you are experiencing a very similar issue.

[Thank you for reviewing our manuscript. As mentioned in our introduction travel distance following complex surgery has been associated with inferior outcomes, particularly for readmission rates. The pick-up rate of early complications, avoiding the need for readmission, may be less in areas further away from the main treatment centre. Additionally, patients who live further from tertiary referral centres may present to local hospitals which may inadvertently lead to higher rates of readmission particularly if clinical decisions do not involve the primary operating surgeon with more experience. This work is particularly important with the recent introduction of revision knee networks in England in April 2022 where a cohort of patients will be expected to travel long distances for management. We have included this additional text in the introduction of the manuscript.]

VERSION 2 - REVIEW

**Reviewer**	**3**
**Name**	**Scandrett, Katie**
**Affiliation**	**University of Birmingham**
**Date**	**27-Nov-2024**
**COI**	**None**

Thank you for opportunity to review this interesting and very well-written submission which was an enjoyable read. I have a few queries regarding some aspects of the methodology that require clarification. Please see my comments below.

Major comments

Was the “Drive time and Miles reference file” taken directly from the UK Department of Transport or was this dataset generated by the authors? This is unclear from the methods section. Perhaps a brief description of how these travel times were calculated (either by the Department of Transport or the authors) might be useful, even if this detail is given in an appendix. For example, use of the TRACC software and Dijkstra’s algorithm to find the shortest distance for travel between the graph networks is mentioned in the referenced document, which is presumably the method used to calculate the travel times for the model presented in table 3.

As already mentioned by reviewer 1, it is unclear why the travel times were split into quintiles. Why was continuous travel time not used? The included reference is a reference for another paper that has also used quintiles to stratify travel times, but no explanation of why this method was used is given. Usually, categorisation of continuous predictors leads to loss of information and is not advised unless there is a clinically meaningful cut-off that is used to partition the data.

There is no mention of how missing data was dealt with in the method section. From the code in the supplementary material, it is clear that some imputation has been conducted (for example, use of imputation using median values for IMD score). Any imputation and reasoning for the choice of imputation technique needs to be described in the methods section. Additionally, a large number of patients (n=3,330) were excluded due to ‘missing/differing Provider Site Code and LSOA’. What does this mean; were distances for the LSOA and Provider Site Codes not included in the travel times dataset?

The analysis includes patients treated at 35 hospital sites run by 16 trusts. Therefore, I question whether a method to account for the clustered (and longitudinal) nature of the data should be considered.

Given the outcome of interest is hospital readmission (and mortality within 90 days), why was a modelling technique for survival data not considered? This approach could also take competing risks into account.

No sample size calculation has been presented and although the analysis includes 1,516 patients, there were only 111 patients who were readmitted within 30 days and there are even fewer events for death within 90 days. Therefore, the analysis is likely to be underpowered. This should at least be mentioned as limitation.

In the results section, it is mentioned that there was no association between distance and the outcomes. However, in tables 2 and 3, there appears to be an increased odds of readmission within 30 days for those in Q2 compared to Q1 after adjustment.

Minor comments

Why is the distance the patient has travelled described as an intervention in the abstract? Would exposure be more appropriate?

The main outcome is emergency readmission within 30 days rather than the association between travel distance and emergency readmission within 30 days.

Please include the odds ratio which describes the relationship between distance and the outcome in the abstract.

Which linking method was used to link the ONS data and the HES data? Please include detail of this in the methods section.

The Journey Time Statistics document does not appear to be in the supplementary material section.

Were the centroids of the LSOA and the hospital post codes used when calculating the distances?

Why were patients excluded if the procedure laterality was not specified?

Which comorbidities and characteristics of initial presentation were included in the models? Please include detail of these covariates in the methods section.

Please include in the table footnotes which covariates the odds ratios have been adjusted for.

Please include in the results section the number of hospitals and trusts that were included in the analysis.

Please include in the results section how many events there were for the secondary outcomes.

The Spearman's rank correlation coefficients are not included in the boxplots.

It seems that off peak driving distance was used for the sensitivity analysis from the code. Please include detail of this in the methods section.

The Shapiro Wilk test is a test for normality. How does this provide information about multicollinearity?

VERSION 2 - AUTHOR RESPONSE

Reviewer: 3
Miss Katie Scandrett, University of Birmingham
Comments to the Author:
Thank you for opportunity to review this interesting and very well-written submission which was an enjoyable read. I have a few queries regarding some aspects of the methodology that require clarification. Please see my comments below.

REPLY: Thank you for these positive comments. We have addressed the specific points below.

Major comments

Was the “Drive time and Miles reference file” taken directly from the UK Department of Transport or was this dataset generated by the authors? This is unclear from the methods section. Perhaps a brief description of how these travel times were calculated (either by the Department of Transport or the authors) might be useful, even if this detail is given in an appendix. For example, use of the TRACC software and Dijkstra’s algorithm to find the shortest distance for travel between the graph networks is mentioned in the referenced document, which is presumably the method used to calculate the travel times for the model presented in table 3.

REPLY: Thank you for highlighting this omission. I have updated the methods section accordingly. Please see lines 177 to 280.

As already mentioned by reviewer 1, it is unclear why the travel times were split into quintiles. Why was continuous travel time not used? The included reference is a reference for another paper that has also used quintiles to stratify travel times, but no explanation of why this method was used is given. Usually, categorisation of continuous predictors leads to loss of information and is not advised unless there is a clinically meaningful cut-off that is used to partition the data.

REPLY: Thank you for your comment. We used categorisation to aid interpretability for clinicians and patients reading the work. However, we agree this will likely lead to loss of information. We repeated our analyses with additional septic patients in the cohort. We did this because we felt this would improve the generalisability of our results. Despite outcomes differing between infected and non-infected patients we thought the case mix was unlikely to be substantially confounded by patient travel distance. We modelled travel distance and time as a continuous variable in our updated multivariable logistic regression models as suggested by the reviewer. We tested for evidence of non-linearity between the exposure and the log of the outcomes using Box Tidwell Tests. Where nonlinearity was found, we modelled the exposure using restricted cubic splines accepting the least number of knots in the model according to model fit statistics.

There is no mention of how missing data was dealt with in the method section. From the code in the supplementary material, it is clear that some imputation has been conducted (for example, use of imputation using median values for IMD score). Any imputation and reasoning for the choice of imputation technique needs to be described in the methods section. Additionally, many patients (n=3,330) were excluded due to ‘missing/differing Provider Site Code and LSOA’. What does this mean; were distances for the LSOA and Provider Site Codes not included in the travel times dataset?

REPLY: Thank you for your comment, this is an important point. We have now repeated the analysis as discussed above and used imputation. I have added reasons for choice of imputation method in the text. Please see lines 358 to 369. Following a repeat analysis using additional data to include both septic and aseptic patients, 5,838 missing travel data was identified out of 16,565 patients before the exclusion criteria was applied. We performed multiple imputation using 5 random imputations using the mice package in R studio. I have included the imputed vs observed values in supplementary material S4. I have demonstrated the way we did this in the methods section of the manuscript. Please refer to lines 631 and 635 which discusses the limitations of using this technique.

The analysis includes patients treated at 35 hospital sites run by 16 trusts. Therefore, I question whether a method to account for the clustered (and longitudinal) nature of the data should be considered.

REPLY: We attempted to account for clustering using a random effects model however this led to convergence issues likely due to numerical instability. Pragmatically, we controlled for the clustered nature of our data between hospital providers through inclusion as a covariate in our modelling. To ensure consistency in our definition of tertiary referral hospitals, only hospitals performing >49 revisions/year were included. These are likely to treat a similar case mix of patients and potentially have similar access to resources within a national healthcare system. This approach allowed us to control for variation across providers. However, we acknowledge it does not fully account for the hierarchical nature of the data with differences in treatment protocols and hospital specialisation among factors which may influence patient outcomes.

Given the outcome of interest is hospital readmission (and mortality within 90 days), why was a modelling technique for survival data not considered? This approach could also take competing risks into account.

REPLY: Thank you for your comment. We did not have access to date of readmission and death, therefore we were unable to model the data in this way.

No sample size calculation has been presented and although the analysis includes 1,516 patients, there were only 111 patients who were readmitted within 30 days and there are even fewer events for death within 90 days. Therefore, the analysis is likely to be underpowered. This should at least be mentioned as limitation.

REPLY: Please see lines 635 to 637. This was an analysis of all data available to us and sample size was set by inclusion criteria. We therefore do not feel a formal sample size calculation is appropriate for this study.

In the results section, it is mentioned that there was no association between distance

and the outcomes. However, in tables 2 and 3, there appears to be an increased odds of readmission within 30 days for those in Q2 compared to Q1 after adjustment.

REPLY: Following earlier comments leading to our decision to model distance as a continuous variable, this analysis has been removed. This section should now be much clearer.

Minor comments

Why is the distance the patient has travelled described as an intervention in the abstract? Would exposure be more appropriate?

REPLY: I have corrected the wording in abstract.

The main outcome is emergency readmission within 30 days rather than the association between travel distance and emergency readmission within 30 days.

REPLY: Change made as suggested. Please see lines 79 to 80

Please include the odds ratio which describes the relationship between distance and the outcome in the abstract.

REPLY: Change made as suggested. Please see lines 86 to 87

Which linking method was used to link the ONS data and the HES data? Please include detail of this in the methods section.

REPLY: We used a unique pseudonymised patient identifier. I have updated methods. Please see line 212/213

The Journey Time Statistics document does not appear to be in the supplementary material section.

REPLY: I have added this Supplementary material S1

Were the centroids of the LSOA and the hospital post codes used when calculating the distances?

REPLY: Yes. I have updated text. See line 227.

Why were patients excluded if the procedure laterality was not specified?

REPLY: We wanted to exclude early re-revisions as these likely represent catastrophic failures with inherently worse outcomes. Laterality was required to identify a revision on the same side. Thus, laterality was essential to conducting the analysis. Imputation of laterality could mean identify a contra-lateral procedure as a re-revision, thus falsely inflating the revision rate. We have added a short sentence

at this point to clarify.

Which comorbidities and characteristics of initial presentation were included in the models? Please include detail of these covariates in the methods section.

REPLY: Please see lines 292 to 331.

Please include in the table footnotes which covariates the odds ratios have been adjusted for.

REPLY: I have added this as a footnote below table 2.

Please include in the results section the number of hospitals and trusts that were included in the analysis.

REPLY: Please see line 412 to 413

Please include in the results section how many events there were for the secondary outcomes.

REPLY: Please see lines 474 to 535

The Spearman's rank correlation coefficients are not included in the boxplots.

REPLY: I have adjusted plots to represent scatterplots. The Spearmans rank correlation is attached in the figure legend.

It seems that off peak driving distance was used for the sensitivity analysis from the code. Please include detail of this in the methods section.

REPLY: Added to lines 283 to 289

The Shapiro Wilk test is a test for normality. How does this provide information about multicollinearity?

REPLY: We agree that this was not clearly described. Shapiro-Wilk's test was only done to test for normality and not multicollinearity. I have updated the text for clarity.

VERSION 3 - REVIEW

**Reviewer**	**3**
**Name**	**Scandrett, Katie**
**Affiliation**	**University of Birmingham**

Date **26-Feb-2025**

COI

I would like to thank the authors for their clear and comprehensive responses to my comments and for updating the manuscript accordingly, which I believe is much improved as a result. I have a few outstanding minor queries as detailed below.

Perhaps a sentence could also be added in the discussion to explain that a time-to-event approach was not possible due to the nature of the outcome data.

Why are models described as ‘pooled’ in the abstract and throughout? This is confusing and I would suggest removing the word ‘pooled’.

Please include the correlation coefficient in the text when referring to the weak correlation between travel distance and age/social deprivation.

The following sentence is unclear: ‘Clinical coding practice within HES is known to vary between trusts but this is unlikely to be vary systematically to bias our findings’. Please revise.

Perhaps the following sentence could be expanded upon: ‘Data linkage was achieved with our clinical dataset following a reproducible workflow’. Is this referring to linking the travel times and the combined HES/ONS datasets?

Is the following ‘Following data linkage, approximately 36% (n = 5,838) of cases did not match with travel data’ referring the linkage between the travel times and the HES/ONS datasets? Please add detail of what linkage is being referred to here.

Why were confounders added incrementally? If there is clinical reasoning that these covariates are confounders, would it not make sense to include them all in the model?

Which covariates were included as categorical? If the aim is to report the association between travel time/distance and the outcomes, then interpretability of the confounding covariates is not relevant.

The figures depicting the travel distance/times and probability of the outcomes are not mentioned in the methods section. Please ensure that it is clear to the reader that use of splines was to create these figures. Were these models adjusted for confounders?

Please ensure that dataset variable names are not included in figure titles (i.e. ‘IMD_score’).

Perhaps it could be mentioned in the discussion that the relationship between travel distance and probability of readmission may be non-linear, as depicted in figure 2. This could potentially explain why the odds ratios do not appear to show an association between the exposure and outcomes.

VERSION 3 - AUTHOR RESPONSE

Perhaps a sentence could also be added in the discussion to explain that a time-to-event approach was not possible due to the nature of the outcome data.

We have added a sentence I lines 424 to 426 of the manuscript with tracked changes to explain this.

Why are models described as 'pooled' in the abstract and throughout? This is confusing and I would suggest removing the word 'pooled'.

We have removed the word 'pooled'.

Please include the correlation coefficient in the text when referring to the weak correlation between travel distance and age/social deprivation.

We have updated the text accordingly

The following sentence is unclear: 'Clinical coding practice within HES is known to vary between trusts but this is unlikely to be vary systematically to bias our findings'. Please revise.

This refers to one of the strengths and limitations bullet points in the manuscript. I have updated this for clarity.

Perhaps the following sentence could be expanded upon: 'Data linkage was achieved with our clinical dataset following a reproducible workflow'. Is this referring to linking the travel times and the combined HES/ONS datasets?

I have updated for clarity. "Data linkage between the HES/ONS dataset and the travel times dataset was achieved using two shared data fields; LSOA and hospital site"

Is the following 'Following data linkage, approximately 36% (n = 5,838) of cases did not match with travel data' referring the linkage between the travel times and the HES/ONS datasets? Please add detail of what linkage is being referred to here.

I have updated the text for clarity. "Following data linkage between the HES/ONS dataset and the travel times dataset, approximately 36% (n = 5,838) of cases did not match."

Why were confounders added incrementally? If there is clinical reasoning that these covariates are confounders, would it not make sense to include them all in the model?

We agree and we have removed this from the text and rephrased to state all co-variates were included in the adjusted model.

Which covariates were included as categorical? If the aim is to report the association between travel time/distance and the outcomes, then interpretability of the confounding covariates is not relevant.

We agree and we have removed this from the text as it is not relevant

The figures depicting the travel distance/times and probability of the outcomes are not mentioned in the methods section. Please ensure that it is clear to the reader that use of splines was to create these figures. Were these models adjusted for confounders?

I have updated the text for clarity "Where implemented, the use of splines was used to create figures depicting the association between travel distance or times and probability of the outcomes. Only adjusted spline models were used to depict these associations"

Please ensure that dataset variable names are not included in figure titles (i.e. 'IMD_score').

I have edited the figures and updated accordingly

Perhaps it could be mentioned in the discussion that the relationship between travel distance and probability of readmission may be non-linear, as depicted in figure 2. This could potentially explain why the odds ratios do not appear to show an association between the exposure and outcomes.

Thank you for this comment. We do not feel this adds to the discussion as non-linear relationships have been presented when found.
